# Inhibitory Activity of Conferone on FAK Activity and Glutamine Metabolism in Human Colorectal Cancer

**DOI:** 10.1111/jcmm.71165

**Published:** 2026-04-29

**Authors:** Hien Thi My Ong, Eda Ates, Jaeyong Jung, Jeong Soo Sung, Jae‐Chul Pyun, Min‐Jung Kang

**Affiliations:** ^1^ Centre for Advanced Biomolecular Recognition Korea Institute of Science and Technology, 5, Hwarang‐Ro 14‐Gil, Seongbuk‐Gu Seoul Republic of Korea; ^2^ Division of Bio‐Medical Science & Technology KIST School, University of Science and Technology, 5, Hwarang‐Ro 14‐Gil, Seongbuk‐Gu Seoul Republic of Korea; ^3^ Department of Materials Science and Engineering Yonsei University, 50 Yonsei‐Ro, Seodaemun‐Gu Seoul Republic of Korea

**Keywords:** colorectal cancer, conferone, FAK inhibition, glutamine metabolism suppression, molecular docking analysis

## Abstract

Colorectal cancer (CRC) is a major global cause of death, with metastases and chemotherapy resistance contributing to poor outcomes. To identify natural compounds with anticancer potential against CRC and elucidate their action mechanisms, the cytotoxicity of 37 natural compounds was evaluated against the HCT116, leading to the identification of conferone as the lead candidate. Its anti‐migratory and anti‐invasive effects were evaluated in HCT116, Colo205, and SW480 cells. The interactions between conferone and focal adhesion kinase (FAK) were assessed through protein expression analysis and molecular docking. Glutaminolysis regulation was determined by LC–MS/MS, and related enzyme levels were detected by western blotting. Conferone inhibited migration and invasion in all three CRC cell lines, though it showed limited anti‐proliferative activity. At 10 μM, conferone reduced FAK and p‐FAK (Tyr397) protein levels, reversing the epithelial‐mesenchymal transition. Docking analysis confirmed direct FAK binding and predicted inhibition of its phosphorylation, with greater affinity than the FAK inhibitor 1,2,4,5‐benzene tetramine tetrahydrochloride. Conferone also downregulated glutaminase and glutamate–ammonia ligase, increasing glutamine and decreasing glutamic acid. Additionally, it suppressed c‐raf phosphorylation and reduced c‐Myc expression, blocking glutaminolysis‐driven metabolism. These findings highlight conferone as a potential therapeutic agent that targets FAK, alters metabolic reprogramming, and impedes CRC progression.

## 1 Introduction

Colorectal cancer (CRC) typically begins in a section of the large intestine (colon) or rectum and usually arises from small clumps of cells called polyps. Although polyps are initially benign, they often progress to colon cancer over time. According to the World Health Organization, CRC is the third most common cancer, with 1.9 million new cases reported in 2021 [1]. The three major histological subtypes of CRC are adenocarcinoma (AC), mucinous adenocarcinoma (MAC), and signet ring cell carcinoma (SRCC) [2]. The prognostic or predictive value of CRC varies at different stages and among the different subtypes. SRCC has a poor prognosis [3], while the prognosis of MAC remains unclear, with varying patient outcomes [4, 5]. Patients with AC generally have a much better prognosis than those with MAC or SRCC [6]. Although surgical approaches, chemotherapeutics, immunotherapy, and radiation are widely used, their efficacy varies due to the complexity of malignancies.

The development of anticancer drugs has markedly improved patient health outcomes over the last decade. However, the majority of patients are diagnosed at an advanced stage of CRC, and many who initially respond to treatment eventually develop resistance to current medications [7]. Given the existing resistance to chemotherapeutic drugs and the undesirable side effects of alternative methods, the development of highly effective medications is imperative to CRC treatment and to considerably reduce clinical challenges. Natural compounds have long been studied in the search for new anticancer agents, either as drugs against CRC or as agents targeting molecular mechanisms in cancer signalling pathways [8, 9]. Plant‐derived drugs with various chemical structures, such as alkaloids, unsaturated fatty acids, polysaccharides, terpenoids, and polyphenols, have been reported to have anti‐CRC benefits in clinical treatment [10]. The preferred first‐line treatment for CRC is oxaliplatin; however, resistance often develops shortly after treatment begins, leading to treatment failure [11].

The progression of CRC is closely linked to various cancer‐promoting mechanisms, among which metabolic reprogramming plays a central role. As glutamine is one of the most abundant circulating nonessential amino acids, it serves as an important carbon and nitrogen source for rapidly proliferating cancer cells. The metabolic pathway is frequently dysregulated in CRC to sustain biosynthetic demands, redox homeostasis, and mitochondrial energy production through glutaminolysis. Key enzymes in this pathway have been linked to enhanced cell proliferation, survival under stress conditions, and invasive potential. Targeting of glutamine metabolism has therefore emerged as a promising therapeutic strategy in cancer [12].

Beyond metabolic pathways, CRC progression is further driven by integrin‐dependent tyrosine‐phosphorylated proteins, which are key regulators of cancer cell growth, survival, and metastasis [13]. The overexpression of focal adhesion kinase (FAK) is significantly higher in CRC‐liver metastases than in primary tumours [14]. A previous study on prognostic biomarkers for CRC found elevated *FAK* mRNA levels in advanced‐stage CRC tumour tissue arrays [15]. Based on this scientific background, targeting FAK and its inhibitory response to active natural compounds is likely to gain interest in the development of new drugs for CRC research.

This study aimed to provide first‐hand in vitro evidence that the selected natural product compound inhibits the growth of CRC cell lines and to evaluate its mechanism of action. Furthermore, molecular modelling strategies were employed to identify the crucial fingerprints of protein‐ligand interactions.

## 2 Materials and Methods

### 2.1 Natural Product Compounds, Cell Lines, and Cell Culture

A panel comprising 37 active compounds at a concentration of 5 mM in dimethyl sulfoxide (DMSO) was sourced from the Korea Chemical Bank (Daejeon, Korea). These compounds were selected based on previously validated anticancer bioactivity data from our group, where we did a systematic screening of 1374 natural product compounds from the same library, identifying candidates demonstrating potent inhibition of cancer cell migration and proliferation [16, 17]. The compound library consisted primarily of natural product–derived molecules and their analogues representing multiple chemical classes, including flavonoids, alkaloids, steroids, and phenolic derivatives, which are known to possess a broad range of biological activities, including anticancer properties. The colorectal cancer cell lines HCT116, Colo205, and SW480, and the normal colon cell line CCD18Co were procured from the Korea Cell Line Bank (Seoul National University College of Medicine, Seoul, Korea). The cell lines were cultured in Roswell Park Memorial Institute (RPMI) medium from Cytiva (Marlborough, MA, USA) supplemented with 10% fetal bovine serum (FBS) and 1% penicillin/streptomycin from GIBCO (Waltham, MA, USA). Culture plates were maintained in a humidified cell incubator at 37°C under 5% CO_2_.

### 2.2 Cell Viability Assay

Cell viability was evaluated using an EZ‐cytox assay (DoGenBio, Seoul, South Korea). The cells were seeded into 96‐well plates at a density of 1 × 10^4^ cells/well and cultured for 24 h. The cells were then treated with various concentrations (1, 6.25, 12.5, 25, 50, and 100 μM) of the 37 active compounds for 24 h. Following treatment, 10 μL of the EZ‐cytox solution was added to each well, and the cells were incubated for 1 h in the dark. The absorbance was measured at 450 nm using a Bio‐Rad microplate reader (Hercules, CA, USA).

### 2.3 Wound Healing and Invasion Assays

Wounds of uniform size were created across the confluent cell monolayer using sterilised 200 μL pipette tips. Cell debris was eliminated through thorough washing with 1× phosphate‐buffered saline (PBS) at 37°C, allowing migration into the wound area. Digital images were captured at 0 and 24 h post‐wounding. The ImageJ software (NIH, Bethesda, MD, USA) was employed to measure the wound width at three distinct locations within each well. The percentage of wound closure was determined by dividing the healed wound width by the initial width at 0 h. Each experiment was conducted in triplicate using triplicate wells.

The Matrigel‐invasion assay was performed using Invasion Chambers with 8.0 μm PET Membrane from Corning BioCoat (Glendale, AZ, USA). Cells (1 × 10^4^ cells/well) were suspended in 100 μL serum‐free media and added to the upper chamber, which was coated with Matrigel from BD Biosciences (Franklin Lakes, NJ, USA). The lower cavity of the transwell was filled with 500 μL of 5% FBS medium containing fibronectin (5 μg/mL) as a chemoattractant. After 24 h, cells were fixed with 4% formaldehyde and permeabilised with 100% methanol, followed by staining with Giemsa (Merck, Rahway, NJ, USA) for 15 min at room temperature. The upper chamber was then cleaned using a cotton swab. A fluorescence microscope (Nikon Eclipse TE 2000‐U; Tokyo, Japan) was used to count cells by randomly selecting multiple fields per membrane. The mean number of cells that penetrated the lower chamber was used to calculate cell invasiveness. Each experiment was performed in triplicate.

### 2.4 Western Blot Analysis

Protein samples were separated using one dimensional 12% Tris‐glycine SDS‐PAGE and transferred to nitrocellulose membranes (Bio‐Rad, Hercules, CA, USA). The membranes were blocked by incubating with 1× TBS, containing 0.5% Tween 20 and 5% skimmed milk, for 1 h before washing with 1× TBST. The membranes were then incubated overnight at 4°C in the dark with the primary antibodies diluted at 1:1000 in 5% bovine serum albumin. After three 5‐min washes with TBST, 1:5000 dilutions of horseradish peroxidase‐conjugated secondary antibodies (Beverly, MA, USA) were added to 5% skim milk, and the membranes were incubated for 1 h at room temperature. Blots were detected using the Ez‐Capture MG system ATTO (Atto Co., Tokyo, Japan) and the chemiluminescence SignalFire ECL Reagent (Cell Signalling Technology, Danvers, MA, USA); the relative intensity of the bands was measured using ImageJ. An antibody against GAPDH, obtained from GeneTex (Irvine, CA, USA), was used as the loading control. Antibodies against FAK (#3285), phospho‐FAK (Tyr397, #3284), snail (#3879), glutaminase 1 (GLS1, #56750), glutamate–ammonia ligase (GLUL, #80636), glutamate dehydrogenase (GLUD, #12793), c‐raf (#9422), phospho‐c‐raf (#9427), and E‐cadherin (#3195) were purchased from Cell Signalling Technology (Danvers, MA, USA).

For transfection, COLO205, HCT116, and SW480 cells were plated on 12‐well plates 24 h before transfection at 50% confluence. Lipofectamine RNAiMAX was used following the manufacturer's protocol (Invitrogen, Carlsbad, CA, USA) for transfection. The final concentration of siRNA was 10 nM for targeting the PTK‐2 gene (Bioneer, Daejeon, Korea). Cells were harvested 48 h after siRNA transfection, and 10 μM of conferone was added. Two different conditions were used for conferone incubation. PTK2 siRNA knockdown was continued or discontinued prior to conferone treatment. For stopping transfection, cells were washed using 1× PBS and cultured with conferone‐containing fresh medium. The used siRNA sequence is listed in Table S1. The western blotting method for FAK, phospho‐FAK, c‐raf, and phosphor‐c‐raf was the same as above.

### 2.5 Molecular Docking Analysis

To predict the interactions between Conferone and FAK, a comprehensive protein‐ligand analysis was performed using the molecular docking tool AutoDock Vina. The crystal structure of FAK (PDB ID: 1MP8) was obtained from the Protein Data Bank (PDB), and water molecules, heteroatoms, ions, and original ligands were removed from the structure. Polar hydrogen atoms and Kollman charges were added to proteins. For ligand preparation, the 3D structures of 1,2,4,5‐benzene tetramine tetrahydrochloride (a known FAK inhibitor) and conferone were obtained from PubChem and docked to identify the target protein‐binding site. Autodock Tools 1.5.6 was used to prepare the PDBQT format of the ligands. Docking analysis was performed by placing a grid box on the ligand's preferred binding site to assign coordinates, such as the centre xyz. Finally, the 3D binding interaction was visualised using PyMOL viewer (https://pymol.org), and 2D diagrams of protein‐ligand interactions were generated using Discovery Studio software 5.0 (Biovia, San Diego, CA, USA).

### 2.6 Quantitative Analysis Using Liquid Chromatography With Tandem Mass Spectrometry (LC–MS/MS)

MP Biomedicals (Santa Ana, CA, USA) supplied the L‐glutamine, L‐glutamic acid, α − ketoglutaric acid, and ammonium formate (NH_4_HCO_2_), which were obtained from Sigma‐Aldrich (St. Louis, MO, USA). Liquid chromatography‐mass spectrometry (LC–MS)‐grade water and acetonitrile were obtained from J.T. Baker (Phillipsburg, NJ, USA). Formic acid (FA) was supplied by Thermo Fisher Scientific (Waltham, MA, USA).

Cells were cultured in RPMI medium under standard conditions. Metabolites were extracted using 80% ice‐cold methanol and disrupted ultrasonically for 20 min on ice, with 5 s intervals between treatments. Chloroform was added, and the mixture was shaken for 20 min. Samples were then centrifuged at 14,000 × *g* for 30 min, and liquid‐phase extraction was repeated twice to ensure maximal recovery. The upper layer was collected and freeze‐dried. Samples were stored at −80°C until analysis; before use, the samples were resuspended in 100 μL of 0.2% formic acid (FA) dissolved in distilled water.

Metabolite analysis was conducted using a UHP LC–MS/MS system combined with an LTQ Orbitrap Velos Pro system (Thermo Fisher Scientific). A Jupiter Proteo C18 column (4.6 × 250 mm, 4 μm, 90 Å) from Phenomenex (Torrance, CA, USA) was used for separation. The mobile phase comprised 10 mM NH_4_HCO_2_ and 0.2% formic acid in (A) water and (B) 90% acetonitrile. The gradient conditions were as follows: 0.0 to 2 min = 30% to 100% B; 2 to 7 min = 100% B; 7 to 10 min = 100% to 30% B; 10 to 30 min = 30% B. The flow rate and injection volume were 0.8 mL/min and 10 μL, respectively. The column oven was maintained at 30°C. For alpha‐ketoglutaric acid (a‐KG), the mass spectrometer was set to negative electrospray ionisation and then to positive electrospray ionisation for glutamine (Gln) and glutamic acid (Glu). Selective reaction monitoring was used for the quantitative analytical assays, and a full scan was performed to confirm the fragment ions of the selected precursor ions. The spray voltage was +3.9 kV, and the collision energy was 30 eV. The gradient elution and MS operating conditions are listed in Table 1.

**TABLE 1:** HPLC gradient and MS operating conditions.

Time (min)	Mobile A%	Mobile B%	Flow rate (mL/min)
0	70	30	0.8
0	70	30	0.8
2	0	100	0.8
7	0	100	0.8
10	70	30	0.8
30	70	30	0.8
**Metabolites**	**Scan mode**	**Precursor ions**	**Fragment ions**
Gln	Positive	[M + H]^+^: 147	130
Glu	Positive	[M + H]^+^: 148	130
aKG	Negative	[M‐H]^−^: 145	101

Cells were cultured in L‐glutamine–free medium and subjected to serum starvation in the absence of FBS prior to method validation of metabolite analysis. Cells were washed twice with PBS and harvested by scraping, and aliquots containing cells (0.5 × 10^6^) were prepared for validation experiments. Calibration and quality control (QC) samples were prepared by spiking cell aliquots with Gln, Glu, and a‐KG standards (1–25 μg/mL). Metabolites were extracted using 80% ice‐cold methanol, as described earlier. Validation encompassed various parameters to ensure acceptable precision and accuracy. The average endogenous background value was subtracted from each analysis to enhance measurement accuracy, accounting for any constant background signal that may be present in the system. The limit of detection (LOD), limit of quantification (LOQ), and absolute recovery (%) were calculated using the following equations:

$$\mathrm{LOD}=3.3^{*}\;\left(a\right)\;\left(b\right)$$

$$\mathrm{LOQ}=10^{*}\;\left(a\right)/\left(b\right)$$

where (a) = Standard deviation of the blank response and (b) = Slope of the calibration curve.

$$\text{Absolute Recovery}\;\left(\%\right)=\left(\text{Measured concentration of spiked sample}-\text{Concentration of unspiked sample}\right)/{\text{Spiked concentration}}^{*}\;100.$$

### 2.7 Statistical Analysis

All data are presented as the mean ± standard deviation (SD) of values from triplicate experiments. *p < 0.05 was considered to indicate significant differences. Different symbols were used for **p < 0.01, ***p < 0.001, and ****p < 0.0001. A one‐way ANOVA followed by Dunnett's multiple comparison tests was performed using GraphPad Prism version 10.0.3 (GraphPad Software Inc., La Jolla, CA, USA).

## 3 Results

### 3.1 Conferone Exerts Inhibitory Effects on the Viability, Migration, Invasion, and Epithelial‐Mesenchymal Transition (EMT) of Colorectal Cancer Cells

**FIGURE 1:**
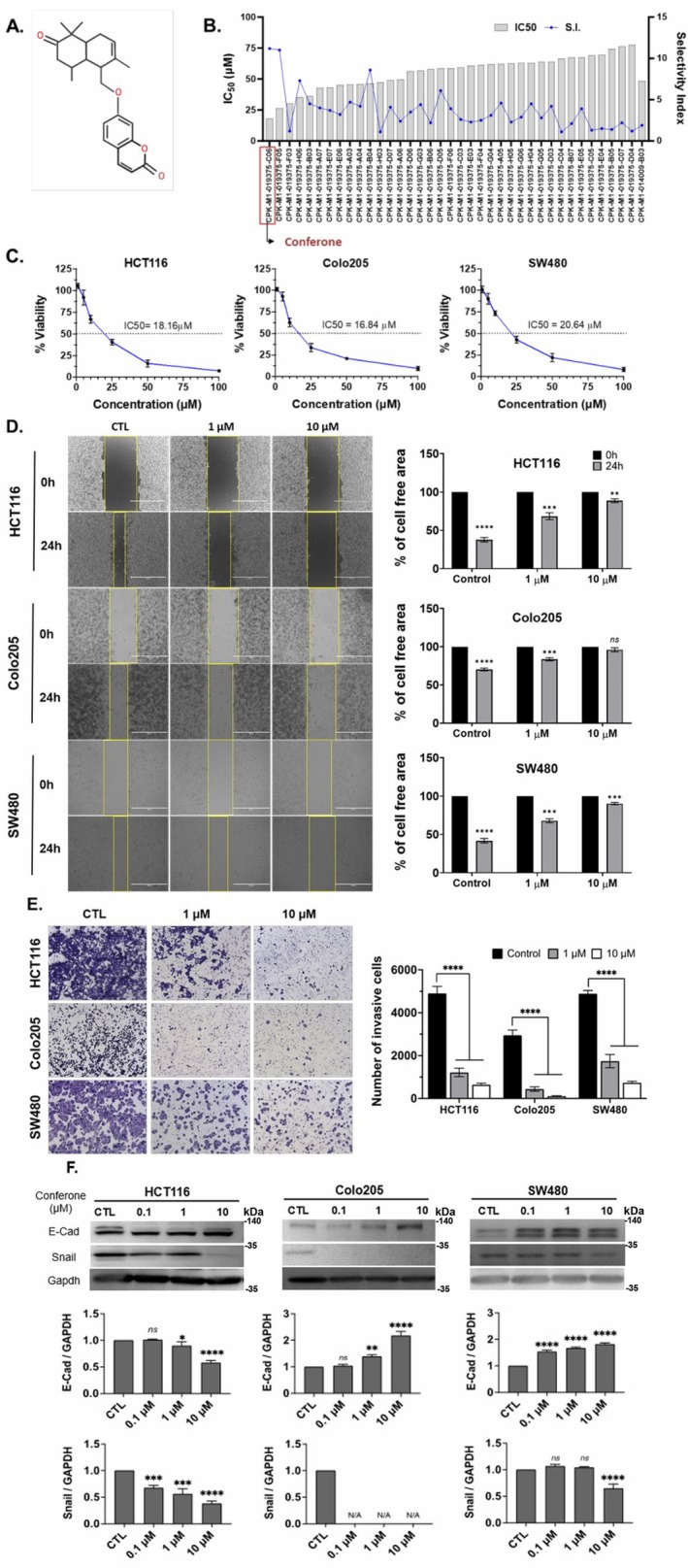
Inhibition of the viability, migration, and invasion of CRC cell lines by conferone. (A) Chemical structure of conferone. (B) Selectivity index of 37 selected compounds in colon cancer cell line (HCT116) vs. normal colon cell line (CCD18 Co). (C) Effect of conferone treatment on the viability of different CRC cell lines. (D) Measurement of wound healing in CRC cell lines after conferone treatment. (E) Assessment of transwell invasion of CRC cell lines after conferone treatment. (F) Protein expression levels of EMT marker, E‐cadherin, and Snail were normalised to those of GAPDH. Bar graphs represent the mean ± SD of values from at least three independent experiments; *p < 0.05, **p < 0.01, ***p < 0.001 and ****p < 0.0001 compared with the control group.

The anticancer effect of conferone (Figure 1A) on CRC cell lines was assessed using the WST assay. Treatment of cancer cells (HCT116) and normal colon epithelial cells (CCD18Co) with increasing concentrations of 37 natural product compounds (1, 5, 10, 25, 50, and 100 μM) for 24 h is depicted in Figure 1B. Among the tested compounds, conferone significantly reduced cancer cell viability in a dose‐dependent manner while exhibiting comparatively lower toxicity toward normal cells, resulting in a selectivity index (SI) of 11.2 (Table 2). The SI serves as a measure of the effectiveness and safety of conferone, indicating how effectively it can inhibit cancer cells compared to normal cells. SI is calculated by dividing the mean IC50 value of normal cells (CCD 18 Co) by the mean IC50 value of cancer cells (HCT116) [18, 19]. Conferone inhibited HCT116, Colo205, and SW480 cell growth in a dose‐dependent manner, with the IC_50_ values ranging from 16.84 to 20.64 μM (Figure 1C). Based on these results and preliminary viability experiments performed with 0.1, 1, 10, and 20 μM conferone, concentrations of 1 μM and 10 μM were selected for subsequent mechanistic studies. Treatment with 20 μM conferone caused a pronounced reduction in cell viability, whereas 10 μM produced a measurable inhibitory effect while maintaining sufficient viable cells for downstream molecular analyses. Therefore, 10 μM was considered an appropriate sub‐IC_50_ concentration, which allowed us to investigate the underlying anticancer mechanisms without excessive cytotoxicity. Additionally, due to the limited availability of conferone, experimental studies were optimised to achieve reliable biological responses and minimise compound consumption.

**TABLE 2:** Cytotoxic activity of natural product compounds on colorectal cancer cells (HCT116) vs. colon normal cells (CCD18 co).

	Compounds	Structure	IC50 (μM)				Selectivity index
			HCT116		CCD18 co		
1	CPK‐M1‐019375‐A03 (Retusin)	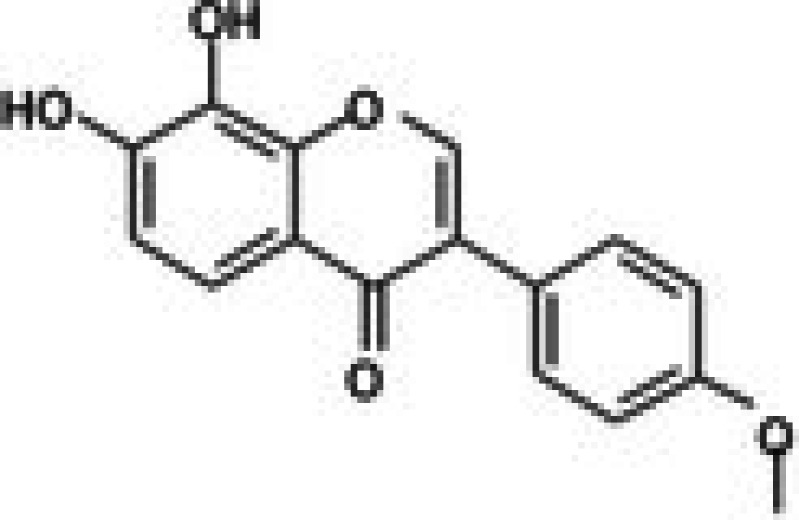	45.73	±1.6	217.15	±56.7	4.7
2	CPK‐M1‐019375‐B03 (Geraldone)	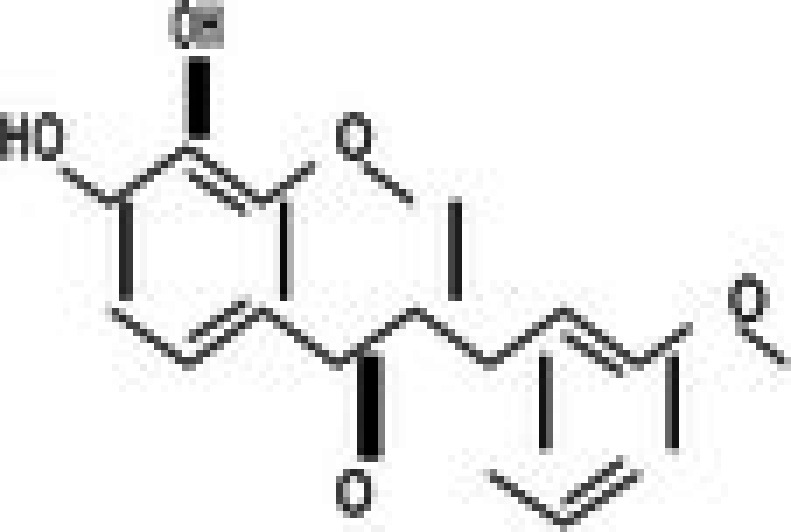	36.30	±2.7	164.44	±37.6	4.5
3	CPK‐M1‐019375‐C03 (Deoxycorticosterone glucoside)	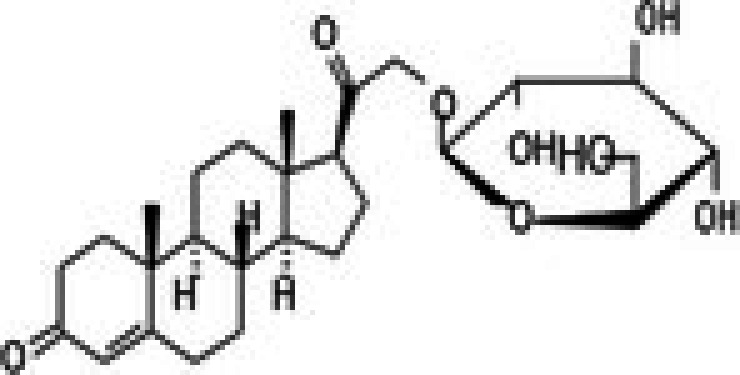	59.38	±3.1	154.93	±37.2	2.6
4	CPK‐M1‐019375‐D03 (2′‐Hydroxydihydrochalcone)	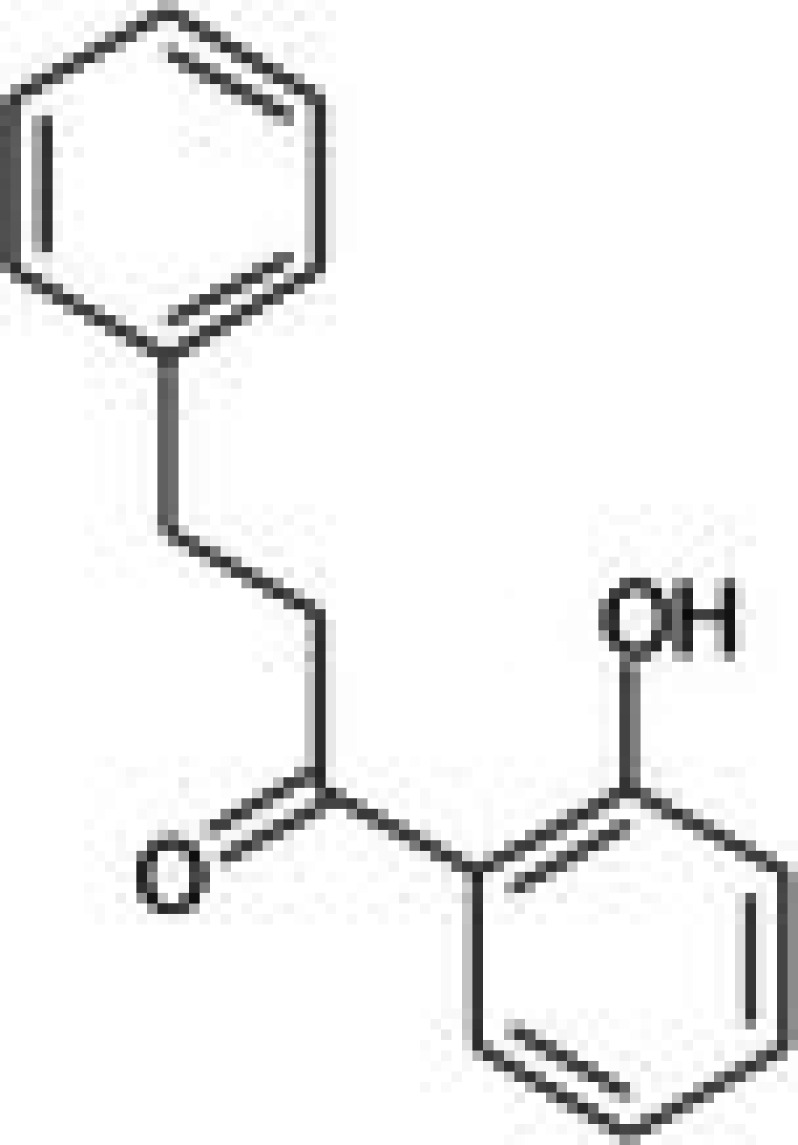	63.82	±3	267.91	±96.5	4.2
5	CPK‐M1‐019375‐E03 (Colchicine)	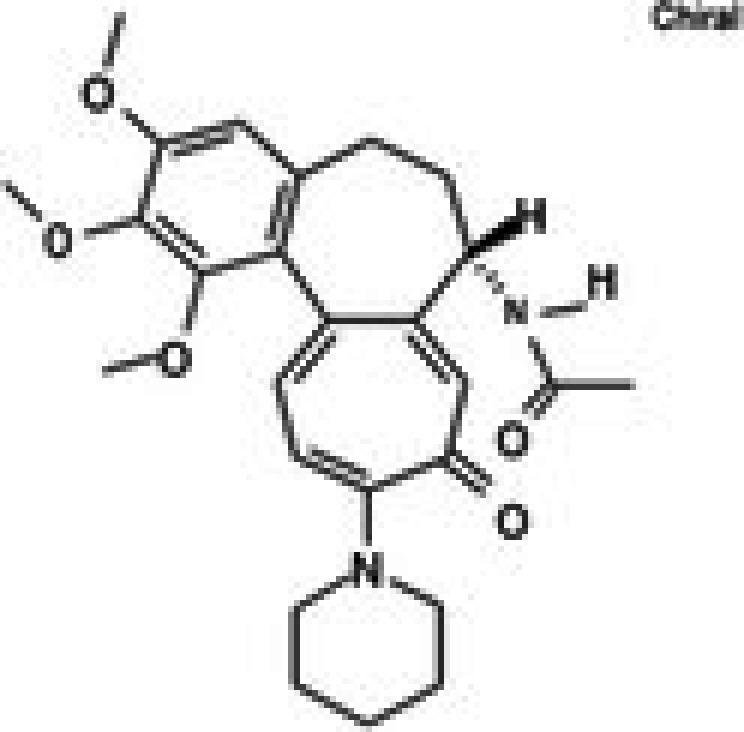	60.91	±4.7	142	±21.2	2.3
6	CPK‐M1‐019375‐F03 (CCT018159)	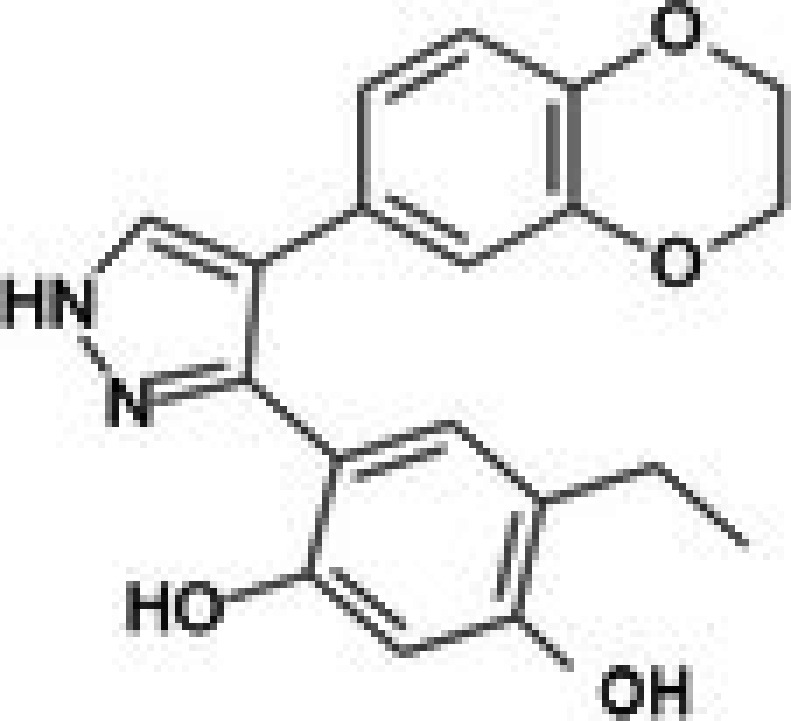	30.34	±1.9	36.56	±3.3	1.2
7	CPK‐M1‐019375‐G03 (GDC‐0068 (Ipatasertib) intermediate)	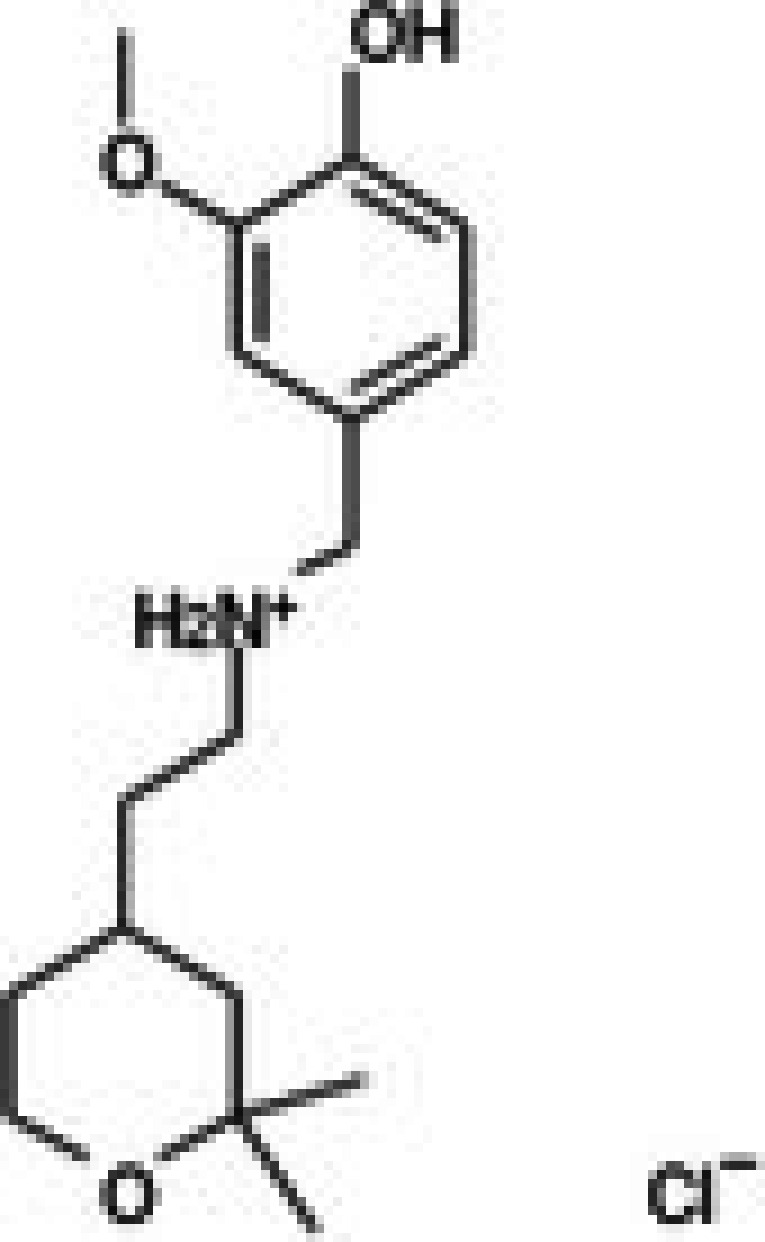	56.86	±2.9	250.17	±67.7	4.4
8	CPK‐M1‐019375‐H03 (6‐Hydroxyformononetin)	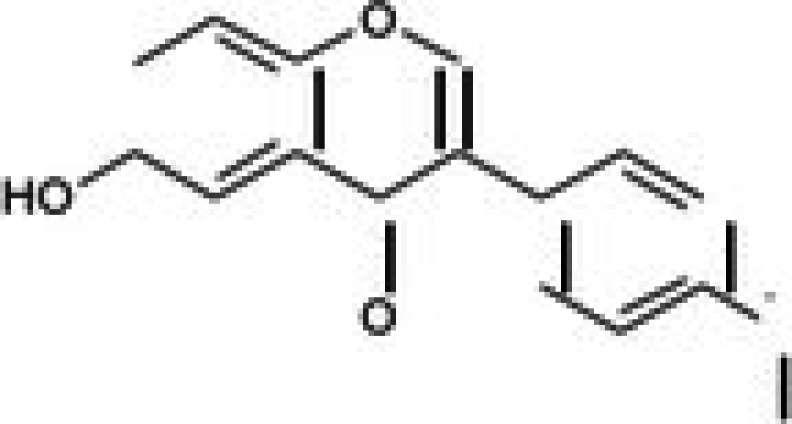	47.26	±2.2	52.47	±3.1	1.1
9	CPK‐M1‐019375‐A04	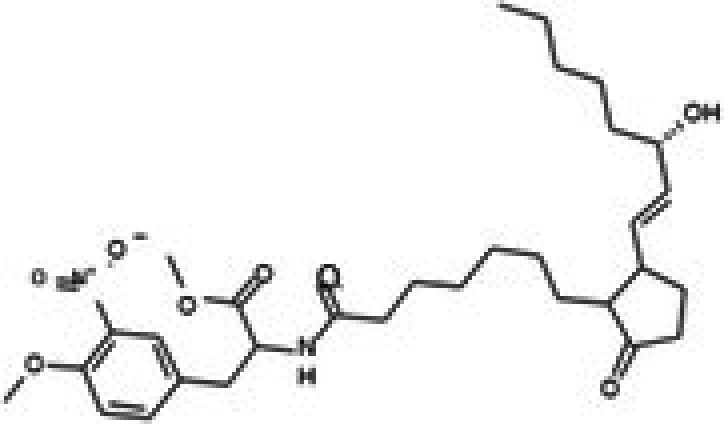	45.85	±4	190.78	±37.3	4.2
10	CPK‐M1‐019375‐B04 (Gitoxigenin)	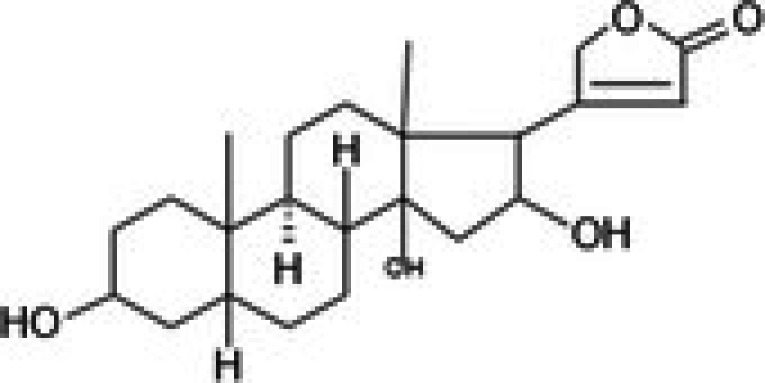	46.69	±3.9	402.88	±151.8	8.6
11	CPK‐M1‐019375‐C04 (2‐aza‐7‐oxaspiro[4.5]decane)	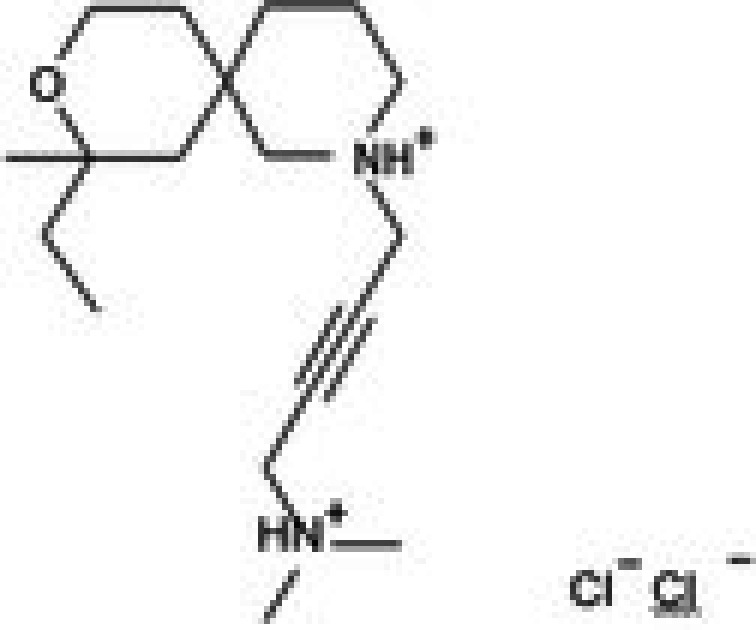	66.75	±1.7	70.45	±2.6	1.1
12	CPK‐M1‐019375‐D04 (Homosyringic acid)	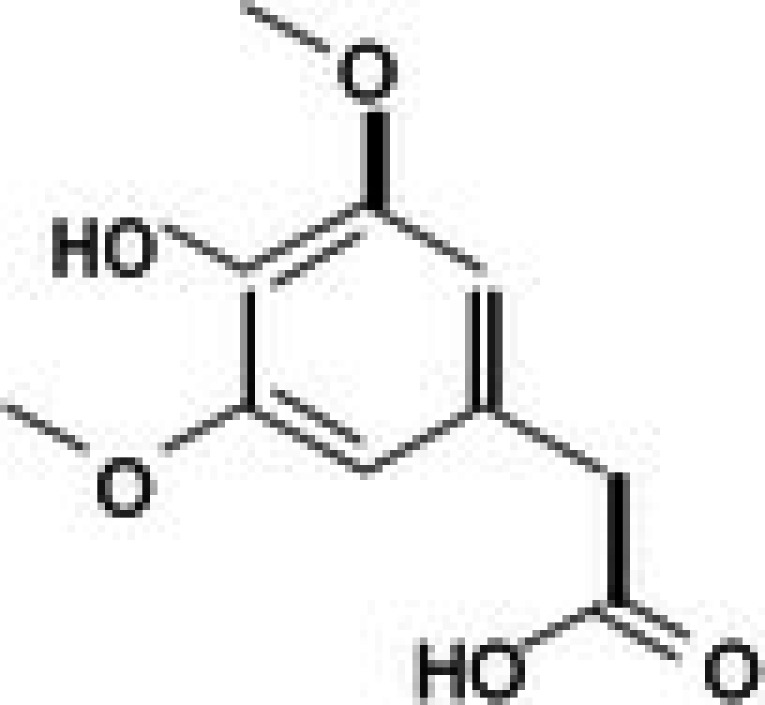	77.46	±3	94.1	±2.5	1.2
13	CPK‐M1‐019375‐E04 (Chelidonine)	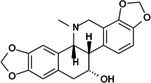	69.56	±3.2	106.01	±6.1	1.5
14	CPK‐M1‐019375‐F04 (Shatavarin IV)	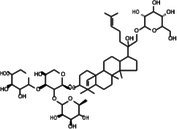	61.18	±1.8	155.56	±17.6	2.5
15	CPK‐M1‐019375‐G04	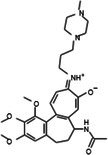	61.78	±2.2	194.07	±46.8	3.1
16	CPK‐M1‐019375‐H04	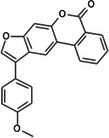	63.03	±3.36	281.35	±79.9	4.5
17	CPK‐M1‐019375‐A05	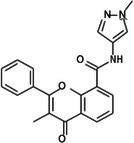	62.47	±3.3	286.33	±65	4.6
18	CPK‐M1‐019375‐B05	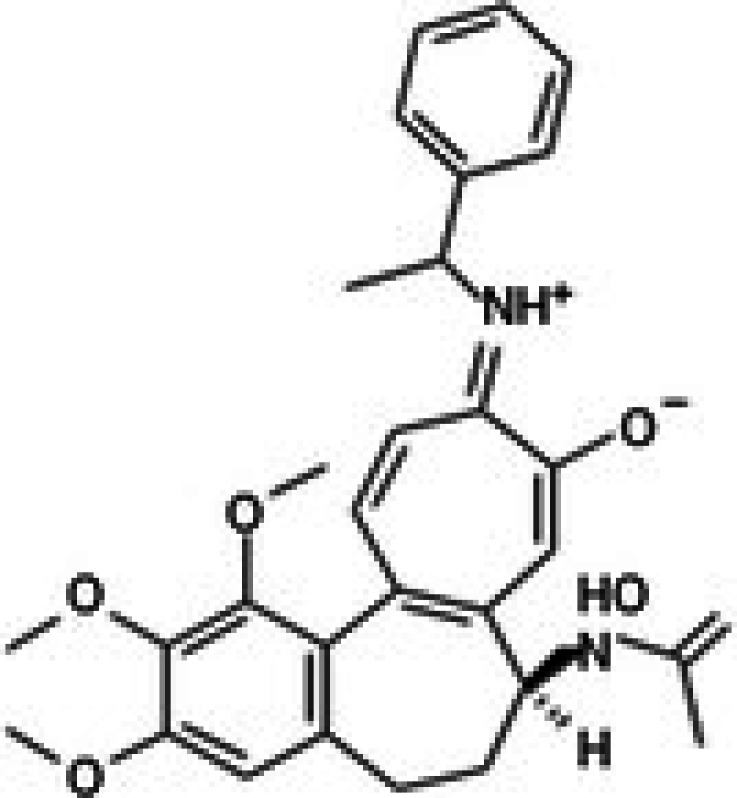	74.24	±3.9	106	±14.2	1.4
19	CPK‐M1‐019375‐C05 (Oridonin)	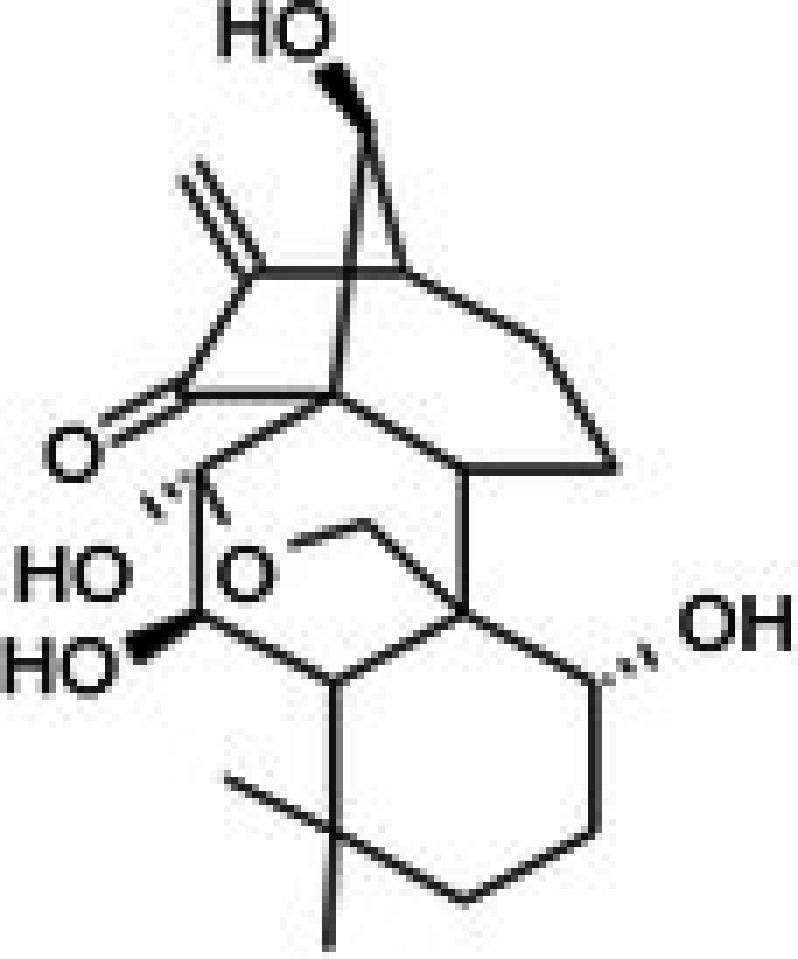	69.27	±4.2	88.17	±7.1	1.3
20	CPK‐M1‐019375‐D05 (6′‐O‐Acetylisoquercitrin)	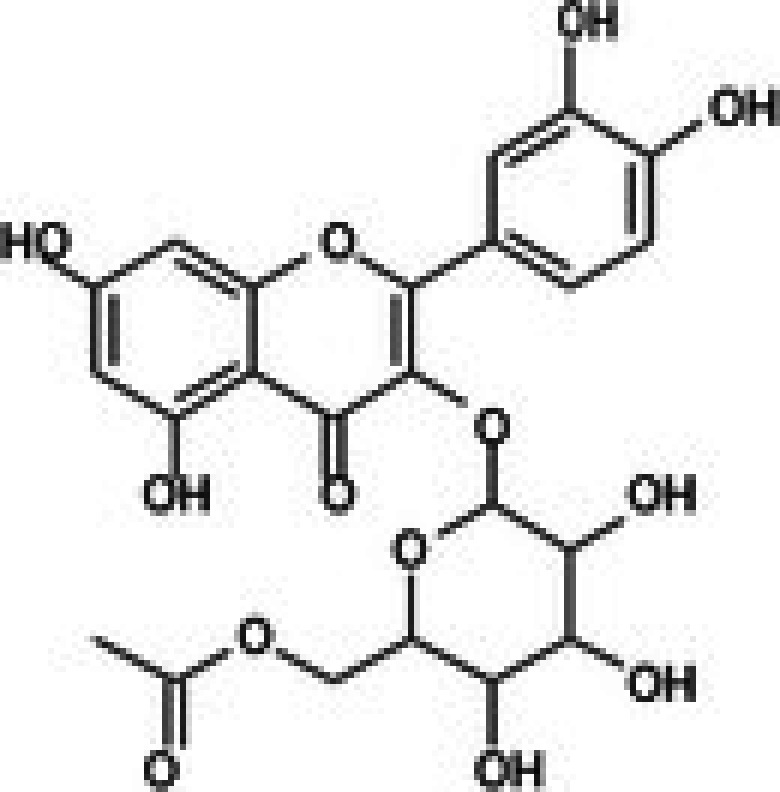	58.72	±3.4	358.09	±168.2	6.1
21	CPK‐M1‐019375‐E05 (Flupentixol dihydrochloride)	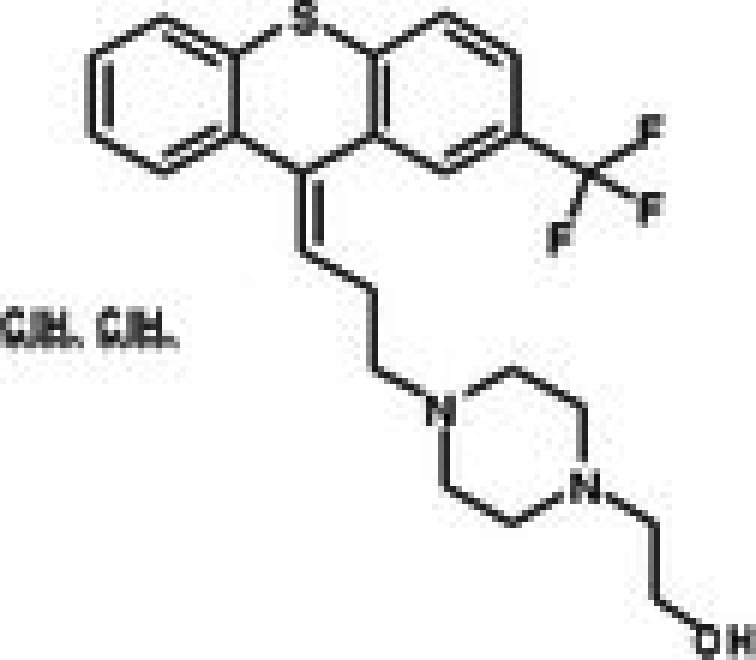	67.49	±1.9	262.68	±70.6	3.9
22	CPK‐M1‐019375‐F05 (Ephedrannin A)	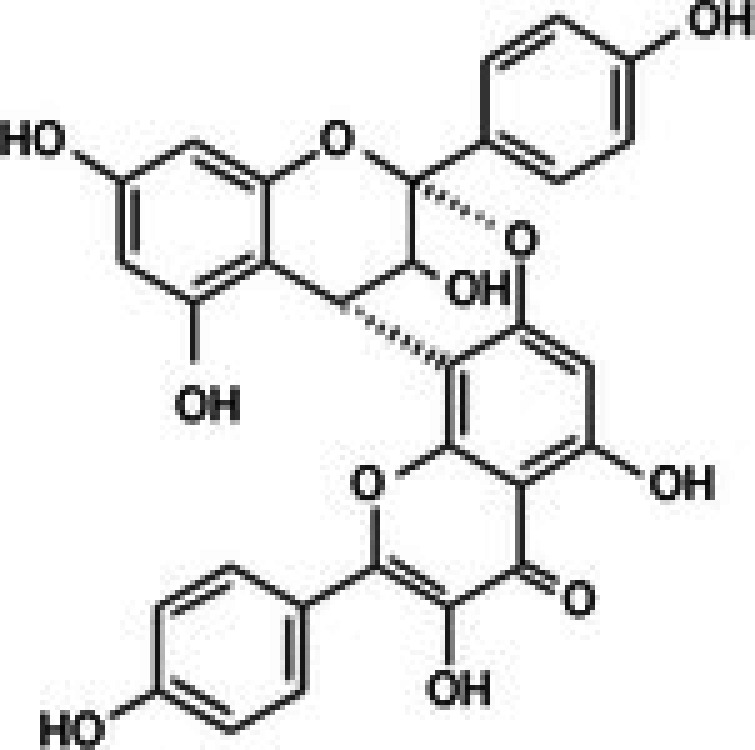	26.52	±2.6	291.73	±80.3	11.0
23	CPK‐M1‐019375‐G05 (Alpha‐dihydrogedunol)	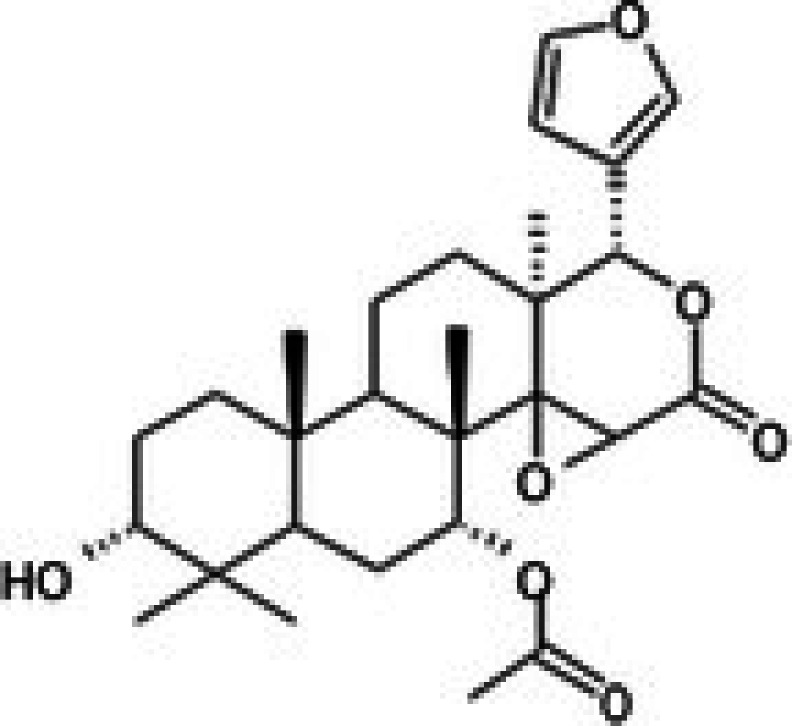	63.74	±4	179.73	±25.2	2.8
24	CPK‐M1‐019375‐H05 (4′‐Demethylepipodophyllotoxin)	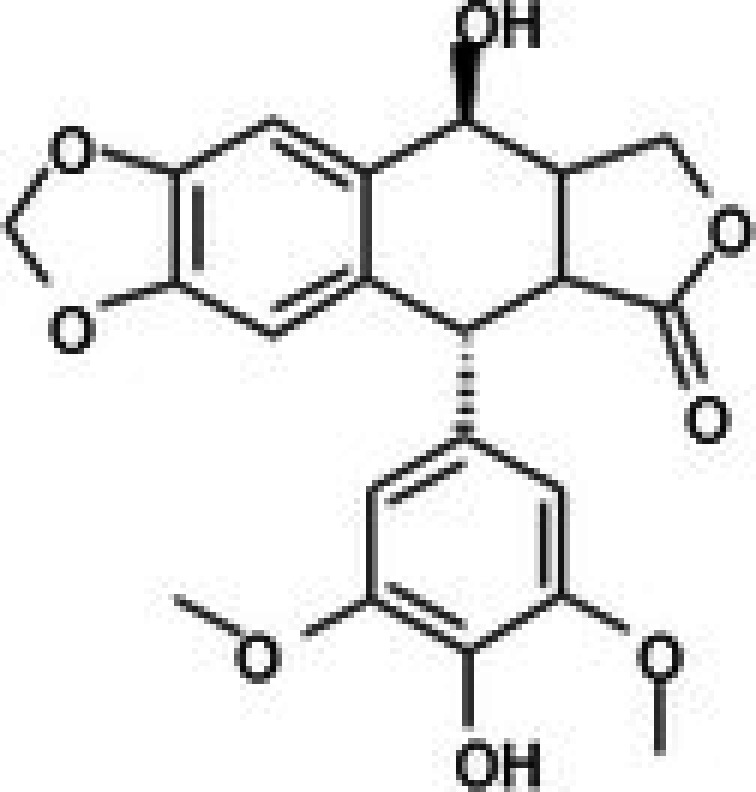	62.63	±2.7	145.28	±17.1	2.3
25	CPK‐M1‐019375‐A06 (m‐Hydroxyphenyl‐tetrahydro‐beta‐carboline acetate)	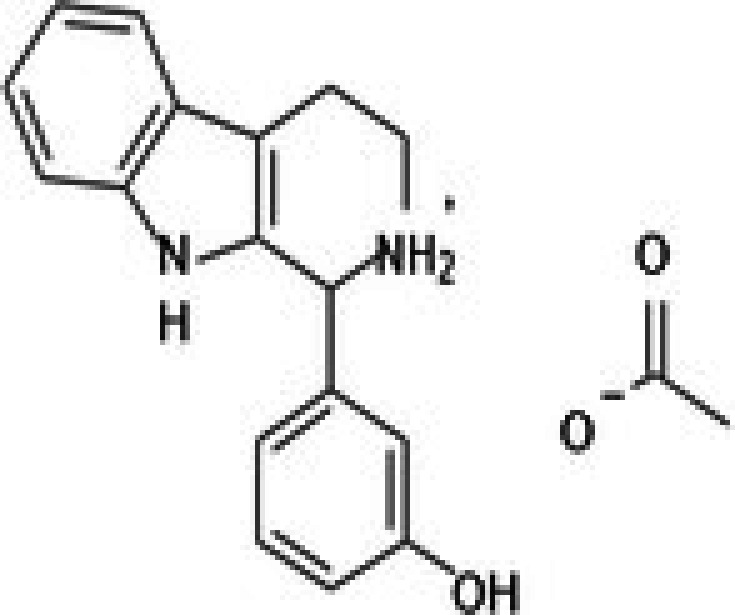	49.75	±3.2	119.51	±6.3	2.4
26	CPK‐M1‐019375‐B06	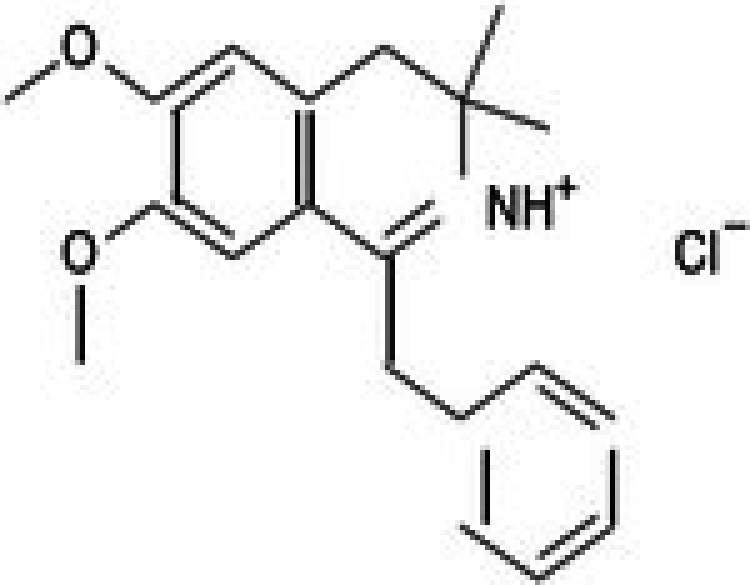	57.86	±3.8	128.79	±10.7	2.2
27	CPK‐M1‐019375‐C06 (Conferone)	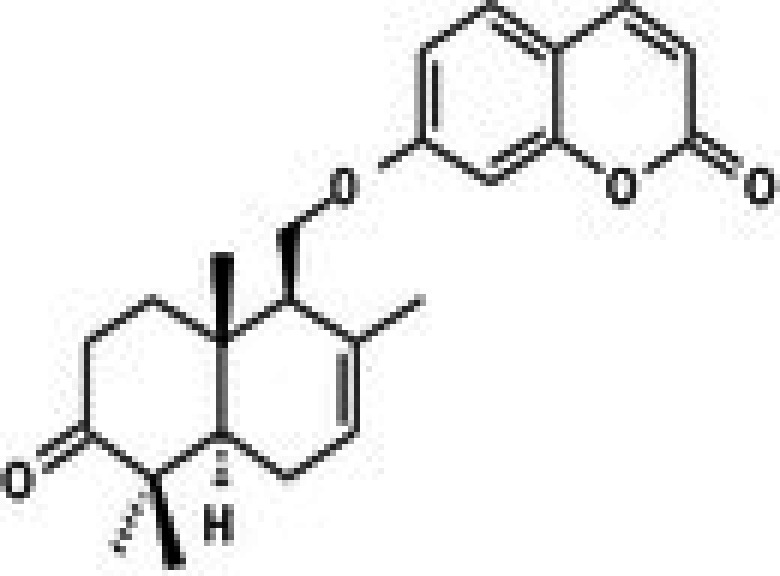	18.16	±0.9	202.92	±29.4	11.2
28	CPK‐M1‐019375‐D06 (Dauricine)	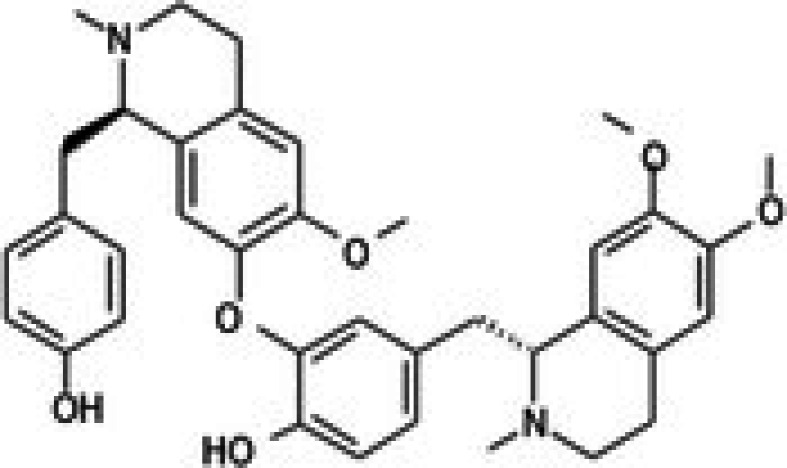	56.39	±3.6	197.45	±38.3	3.5
29	CPK‐M1‐019375‐E06 (Triptolide)	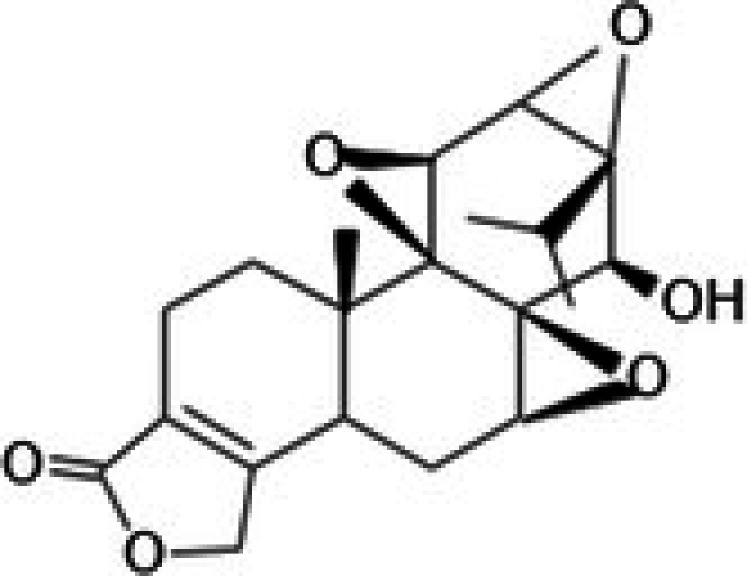	45.23	±1.3	144.23	±7.7	3.2
30	CPK‐M1‐019375‐F06 (Josamycin)	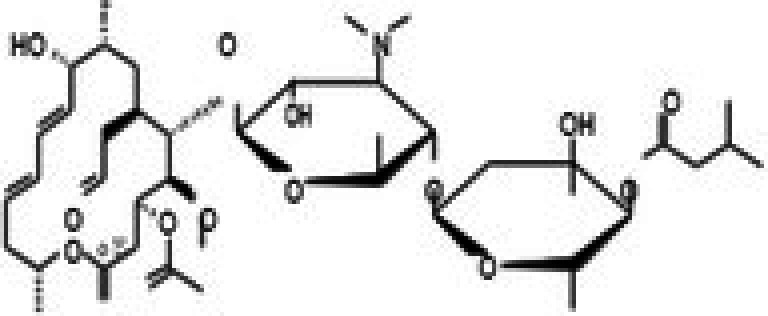	58.76	±4.9	230.15	±31.3	3.9
31	CPK‐M1‐019375‐G06 (PD 102807)	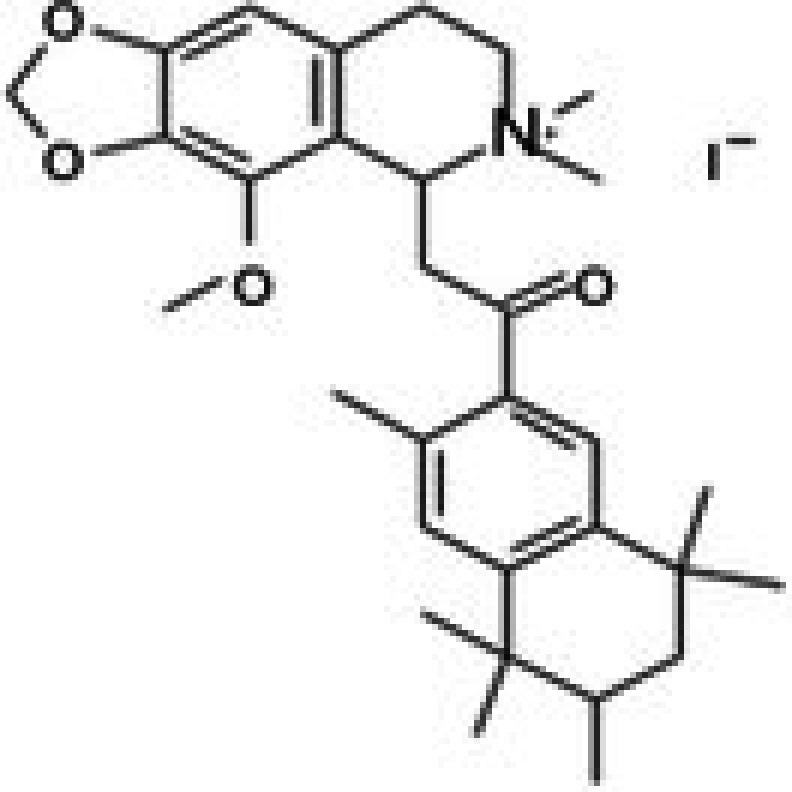	62.98	±3.4	185.39	±27.6	2.9
32	CPK‐M1‐019375‐H06 (7,8‐Dihydroxyflavone)	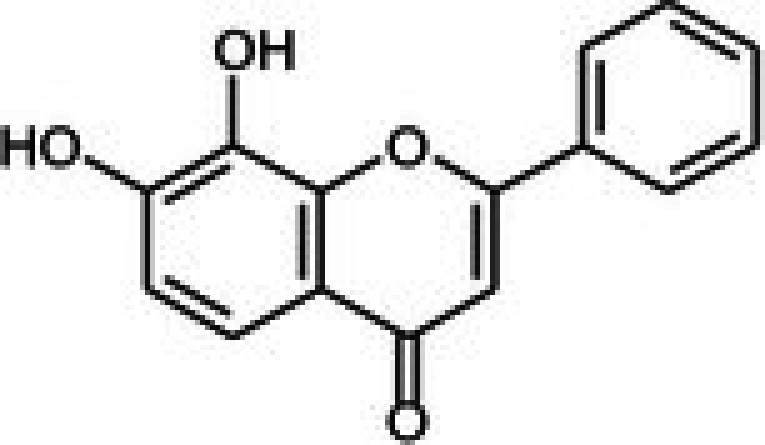	35.24	±1.7	257.57	±65.6	7.3
33	CPK‐M1‐019375‐A07 (Helichrysetin)	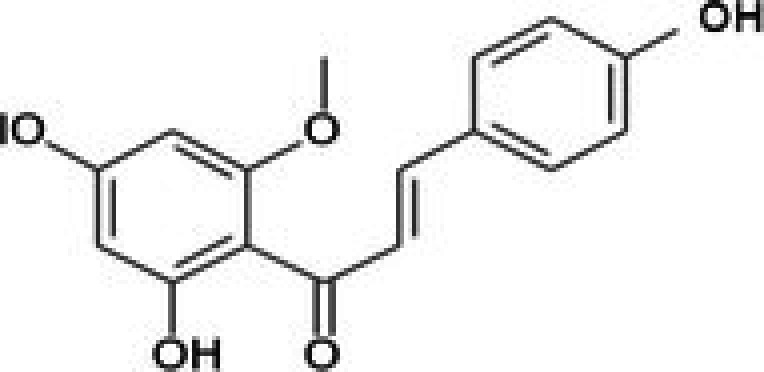	42.89	±2.3	172.06	±24.5	4.0
34	CPK‐M1‐019375‐B07 (1‐Dehydro‐[10]‐gingerdione)	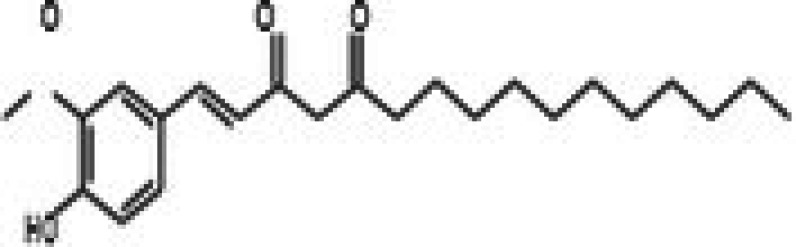	67.34	±4.4	139.25	±19	2.1
35	CPK‐M1‐019375‐C07 (Pyrrolobenzodiazepine)	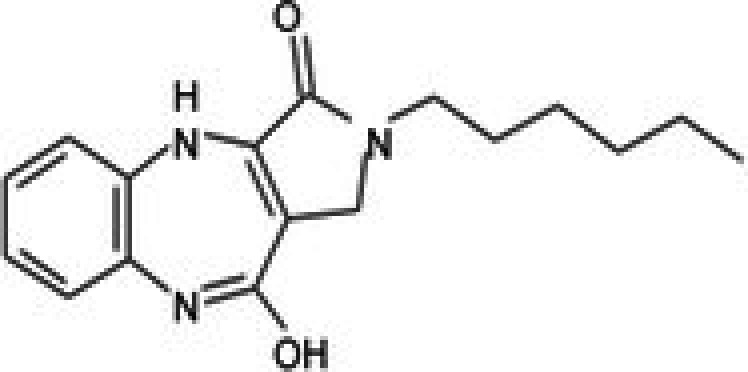	76.73	±3.5	168.35	±24.2	2.2
36	CPK‐M1‐019375‐D07 (8‐Aminomethyl‐7‐hydroxyisoflavone)	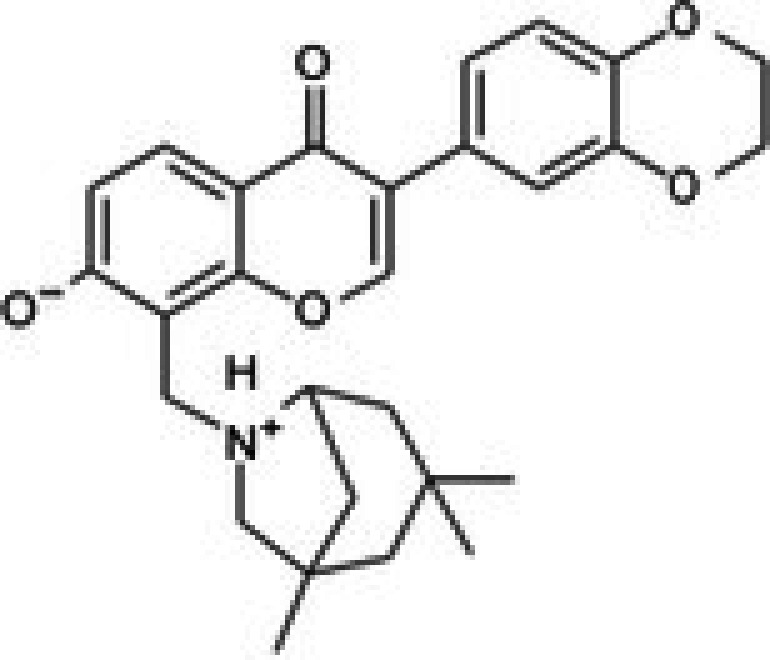	49.07	±3.7	198.75	±30.5	4.1
37	CPK‐M1‐019375‐E07 (Solvent red)	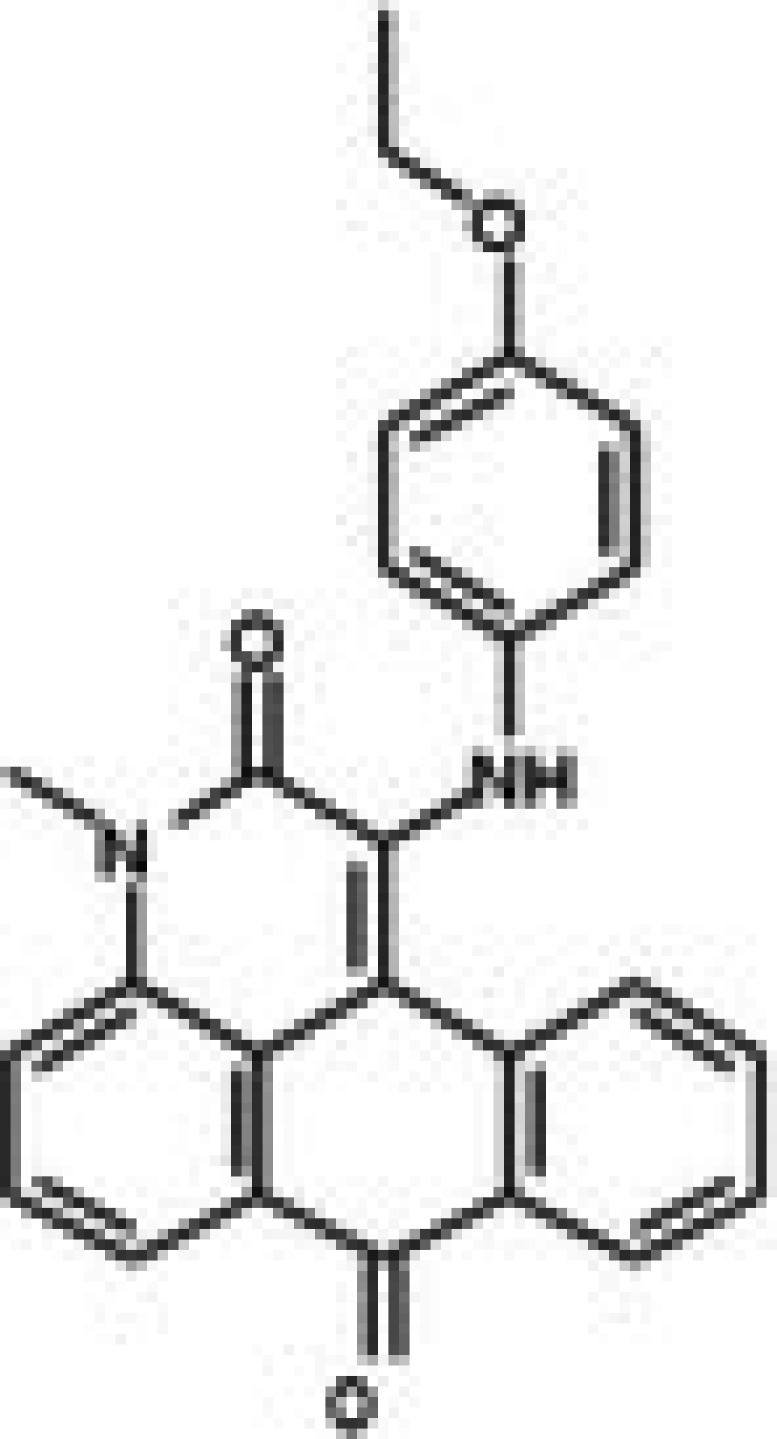	43.29	±2.5	158.37	±18.6	3.7

*Note:* Selectivity Index (calculated as the average of the IC50 value in the normal cell line CCD18Co divided by the IC50 value in the cancer cell line HCT116 obtained in each independent experiment) (Lopez‐Lazaro, 2015; Radha Abbas Hasson and Jawas Kadhim, 2021).

A wound‐healing assay was conducted to explore the impact of conferone on cell migration. Compared to that of the control cells treated with DMSO, conferone treatment markedly suppressed the wound‐healing capability of cancer cells. The extent of cell‐free areas was used to quantify this inhibitory effect. After 24 h of treatment with 1 and 10 μM conferone, the percentages of cell‐free areas in the HCT116 cell line exceeded 70% and 85%, respectively. Similarly, the inhibitory effect on Colo205 and SW480 cell migration at 24 h remained above 70% at both concentrations (Figure 1D). Furthermore, using the Matrigel transwell assay, we demonstrated that conferone treatment significantly reduced the invasive capacity of three CRC cell lines in a dose‐dependent manner. Cancer cells exhibited significantly lower infiltration through the membranes than those treated with DMSO (Figure 1E). These findings underscore that conferone inhibits colon cancer cell migration and invasion in a dose‐dependent manner.

Western blotting was employed to study the impact of conferone on the EMT protein markers associated with metastasis. The results revealed a reduction in the levels of EMT markers, characterised by increased levels of E‐cadherin and decreased levels of Snail (Figure 1F). These changes suggest that conferone effectively inhibits cancer progression by suppressing the EMT. Overall, these findings indicate that conferone markedly reduces the viability, migration, and invasion of HCT116, Colo205, and SW480 colon cancer cells.

### 3.2 Conferone Treatment Suppresses FAK Expression in CRC Cells and Its Interplay With Protein Kinase Pathways

High expression of FAK is linked to colon cancer, and targeting FAK and its binding partners represents a promising therapeutic approach for CRC [20]. FAK serves as a crucial upstream regulator of the mitogen‐activated protein kinase (MAPK), influencing diverse cellular processes downstream. To elucidate the mechanism of conferone action, we investigated its effects on key mediators within the Raf signalling pathway.

**FIGURE 2:**
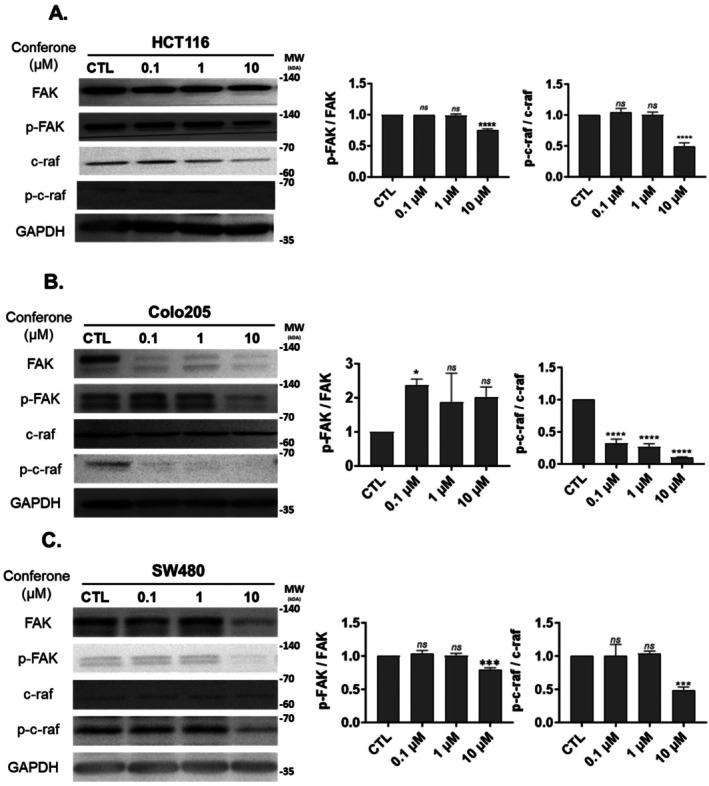
Expression levels of FAK, phosphorylated‐FAK, and related MAPK pathway proteins in three CRC cell lines. (A) HCT116; (B) Colo205; (C) SW480. Bar graphs represent the mean ± SD of values from at least three independent experiments; *p < 0.05, **p < 0.01, ***p < 0.001 and ****p < 0.0001 compared with the control group.

Treatment with 10 μM conferone initiated the suppression of phosphorylated Fak and c‐Raf expression levels in HCT116 cells (Figure 2A). A significant reduction was observed in phospho‐c‐Raf expression in Colo205 cells even at 0.1 μM conferone concentration. Additionally, FAK expression significantly decreased at 0.1 μM concentration, suggesting increased conferone efficacy against signalling in Colo205 cells (Figure 2B). However, at higher concentrations of conferone, Colo205 cells did not exhibit any significant decrease in FAK expression. This lack of response at elevated concentrations may be attributed to potential compensatory mechanisms within the cells, where alternative signalling pathways could be activated to counteract the effects of conferone. Treatment with 10 μM conferone markedly reduced the phosphorylated FAK to FAK ratio expression in SW480 cells, consequently inhibiting phospho‐c‐Raf (Figure 2C). These findings collectively indicate that conferone effectively suppresses FAK phosphorylation, thereby modulating downstream c‐Raf signalling inactivation. To further understand the relation between FAK and c‐Raf signalling pathways, the cells were treated with FAK siRNA in the presence or absence of conferone. Figure S1 shows that the FAK knockdown reduced the levels of both FAK and c‐Raf. However, additional treatment with conferone did not further significantly decrease c‐Raf and phospho‐c‐Raf levels. This regulatory mechanism highlights the inhibitory effect of conferone on the FAK‐c‐Raf signalling pathway in colon cancer cells.

### 3.3 Identification of the Protein‐Ligand Binding Site That Inhibited FAK Expression

**FIGURE 3:**
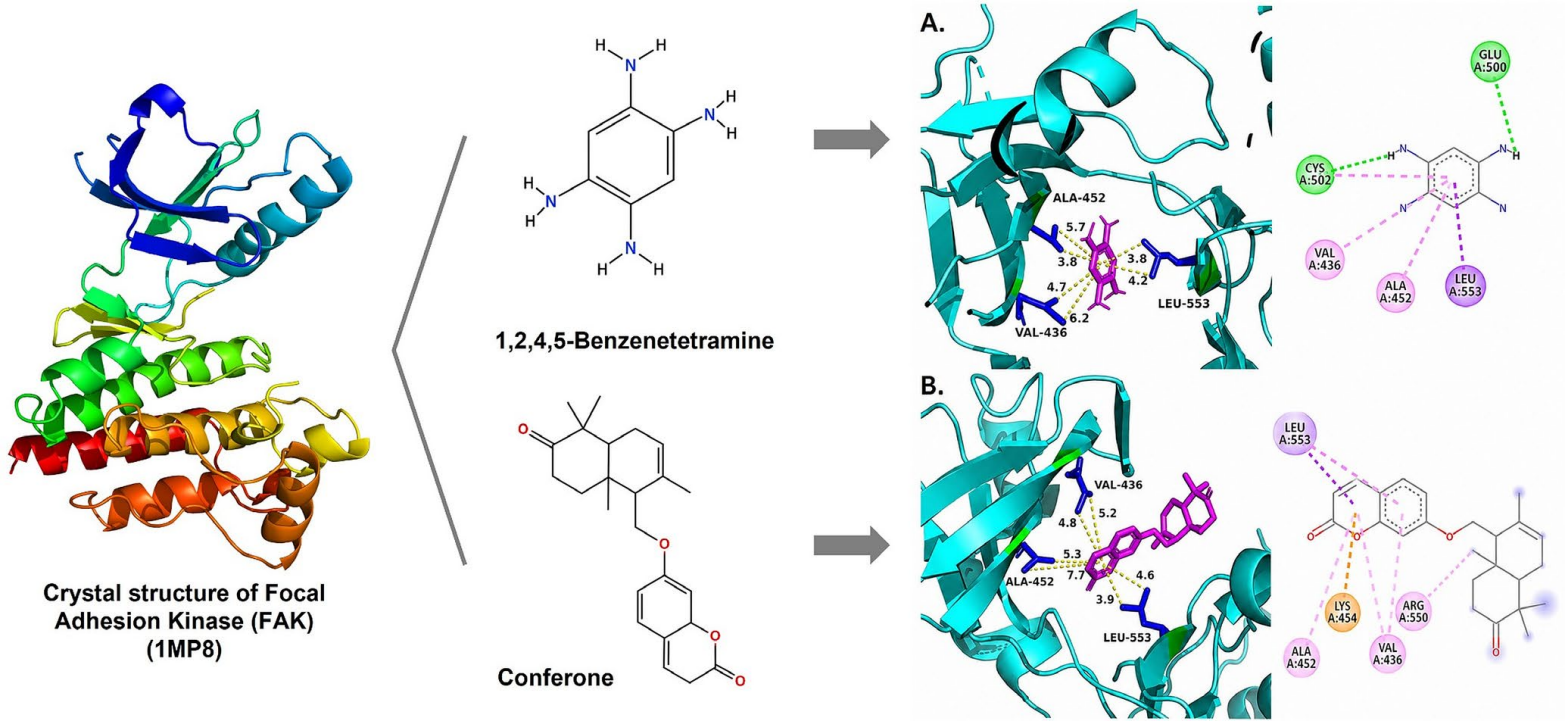
Structure‐based model and docked binding with key residues of FAK and the two ligands. Representative compounds: (A) FAK‐inhibitor (1,2,4,5‐benzene tetramine tetrahydrochloride) and (B) conferone.

Molecular docking studies were conducted to investigate the interactions between conferone and FAK as the target protein. The docking results of conferone and 1,2,4,5‐benzene tetraamine tetrahydrochloride (a known FAK inhibitor) into the target protein (1MP8) were compared; their binding sites and affinities are summarised in Table 3. Illustrates in Figure 3 the predicted binding interactions in both 2D and 3D diagrams. Conferone exhibited favourable binding affinities (−7.1 kcal/mol). It shares similar amino acid interaction sites with 1,2,4,5‐benzene tetraamine tetrahydrochloride, involving two pi‐alkyl interactions with Ala 452 and Val 436 via an aromatic group and one pi‐sigma bond with Leu 533. In the conferone docking position, there was a pi‐cation interaction with Lys 454 near an aromatic side chain and an alkyl bond with Arg 550. These findings suggest that conferone effectively binds to FAK, similar to the interaction pattern observed with 1,2,4,5‐benzene tetraamine tetrahydrochloride. This molecular docking analysis provides valuable insights into the potential of conferone as an FAK inhibitor for anticancer therapy.

**TABLE 3:** Analysis and molecular docking results of FAK inhibitor (1,2,4,5‐benzene tetramine tetrahydrochloride) and conferone.

Structure from PDB	Compound	Binding affinity (kcal/mol)	Related amino acid residues
1MP8	1,2,4,5‐Benzenetetramine	−4.6	**Leu 533, Ala 452, Val 436**, Cys 502, and Glu 500
	(FAK inhibitor)		
	Conferone	−7.1	**Leu 533, Ala 452, Val 436**, Lys 454, and Arg 550

### 3.4 Conferone Is Associated With Glutamine Metabolism Suppression in CRC

Glutamine metabolism plays a crucial role in supporting the proliferation and survival of cancer cells, as glutamine metabolism serves as a major nutrient source for dividing cells [21]. Therefore, we further investigated the conferone's impact on glutamine metabolism by employing an LC–MS/MS method. MS/MS parameters for the target compounds—Gln, Glu, and αKG—wereoptimised. The multi‐reaction monitoring chromatogram and MS/MS spectra in the full scan of the metabolite standards and those extracted from cell lines are depicted in Additional file 1. This method, with a total runtime of 30 min, is well‐suited for the precise quantification of metabolite molecules in analytical studies.

The samples were spiked with standards ranging from 1 to 25 μg/mL to construct calibration curves, demonstrating linearity, with coefficients of 0.999, 0.991, and 0.994. Method sensitivity was evaluated using the LOD and LOQ, yielding LODs ranging from 0.02 to 0.07 μg/mL and LOQs between 0.07 and 0.2 μg/mL. The intra‐ and inter‐day precisions and accuracies are detailed in Table 4. Absolute recoveries ranged from 100.9% to 104.9%. In complex biological samples such as cell extracts, endogenous metabolites may still be present despite starvation and the washing steps. Residual endogenous levels of the studied metabolites, therefore, may lead to calculated recoveries slightly above 100%. The relative standard deviation (RSD%) of triplicate measurements for all analyses was below 20%, indicating the suitability of the method for quantitative analysis.

**TABLE 4:** Inter‐ and intra‐day precision, accuracy, sensitivity, matrix effects, and absolute recovery for metabolites analysis.

Metabolites	Nominal concentration (μg/mL)	Intra‐day			Inter‐day			Linearity	LOD (μg/mL)	LOQ (μg/mL)	Absolute recovery (%)
		Determined concentration (μg/mL)	Precision (RSD%)	Accuracy (%)	Determined concentration (μg/mL)	Precision (RSD%)	Accuracy (%)				
Gln	2.5	2.6 ± 0.11	4.12	103.81	2.6 ± 0.07	2.56	103.96	1	0	0.1	100.9
	10	10.6 ± 0.58	5.45	105.76	10.5 ± 0.36	3.46	105.18				
Glu	2.5	2.6 ± 0.19	7.26	103.91	2.49 ± 0.08	3.13	99.6	0.99	0	0.1	104.9
	10	10.8 ± 0.29	2.65	107.62	10.61 ± 0.54	5.11	106.07				
aKG	3	3.16 ± 0.05	1.52	105.36	3.17 ± 0.08	2.46	105.67	0.99	0.1	0.2	101.8
	8	8.24 ± 0.32	3.94	102.96	8.03 ± 0.17	2.14	100.33				

**FIGURE 4:**
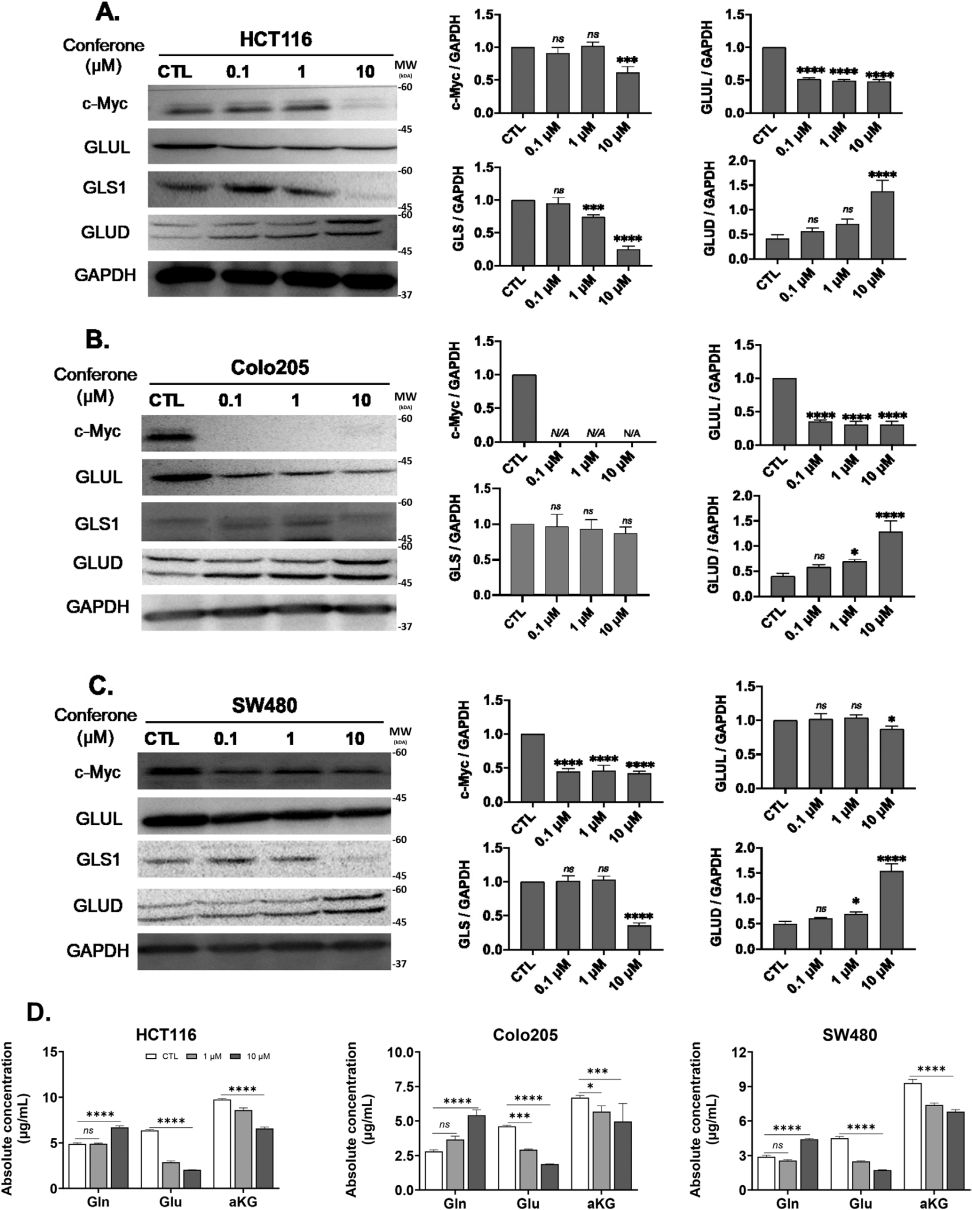
Conferone treatment suppressed glutamine metabolism in the three CRC cell lines. (A–C) Expression levels of c‐Myc, GLS, GLUL, and GLUD were determined using western blot analysis. (D) Levels of Gln, Glu, and alpha‐ketoglutaric acid (aKG) in control and conferone‐treated cells were determined via quantitative analysis. Data are presented as the mean ± SD of values from at least three independent experiments; *p < 0.05, **p < 0.01, ***p < 0.001 and ****p < 0.0001 compared with the control group.

**FIGURE 5:**
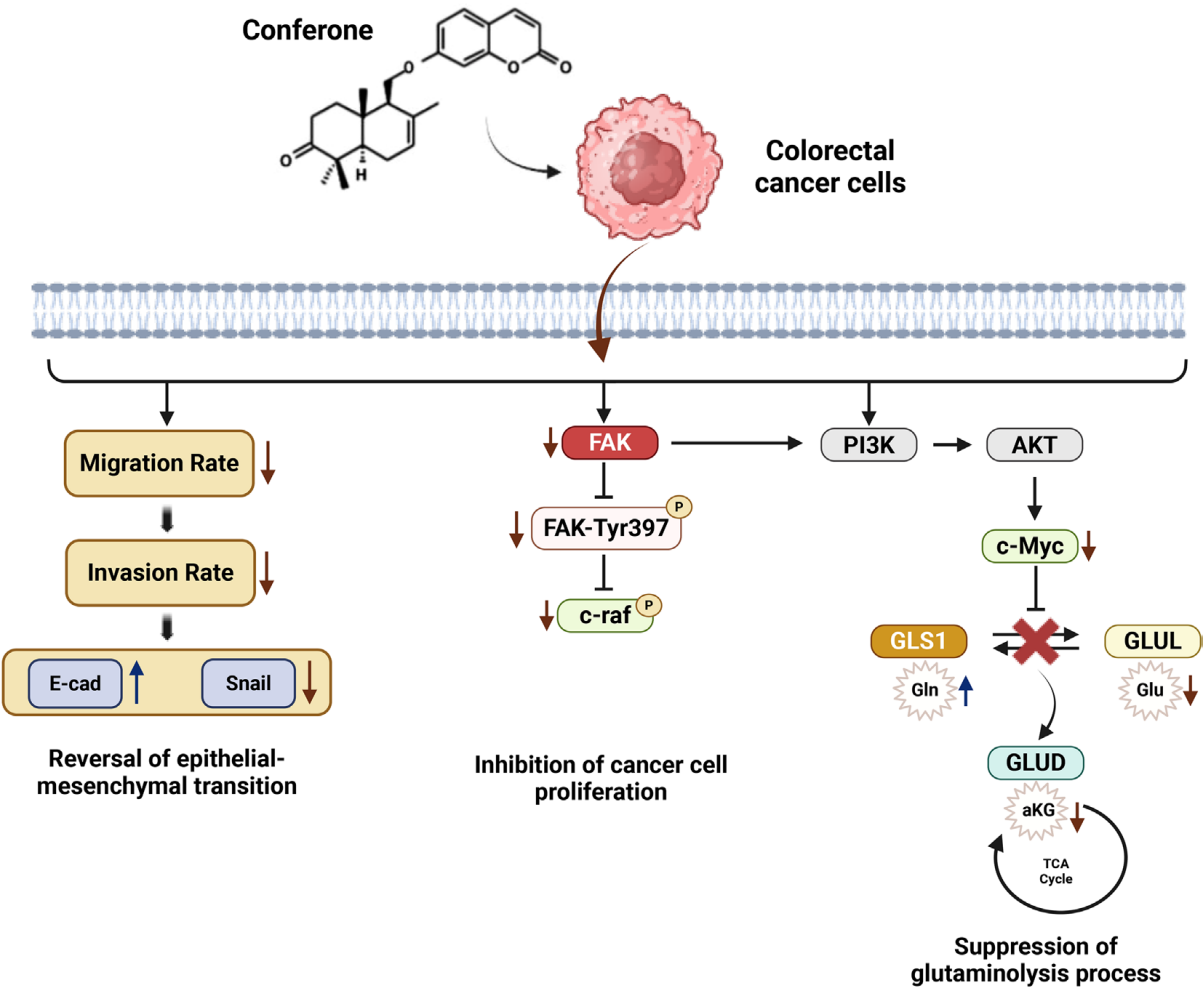
Signalling pathway that promoted the anticancer mechanism of conferone by inhibiting the FAK‐related pathways and glutaminolysis process. Created with BioRender.com.

We further investigated the impact of conferone on the inhibition of glutamine metabolism in HCT116, Colo205, and SW480 cells (Figure 4A–C). After 6 h of conferone treatment, a significant decrease was observed in c‐Myc levels. These results indicated the downregulation of GLS1 and GLUL compared to that in the control group, while GLUD levels were notably increased across all three CRC cell lines. Inhibition of GLS1 and GLUL expression by conferone suppressed glutamine metabolism, leading to reduced glutamate production (Figure 4D). Although GLUD expression increased, it was insufficient to maintain α‐KG production, potentially due to the increased consumption of glutamate by other cellular processes. The anticancer effect of conferone on CRC cells (Figure 5) may thus stem from its ability to suppress the expression of key metabolic enzymes involved in glutaminolysis.

## 4 Discussion

The HCT116, Colo205, and SW480 cell lines collectively provide a robust platform for initial investigations into colon cancer due to their diverse genetic backgrounds. HCT116 carries mutations in tumour suppressor genes (*APC* and *p53*), Colo205 exhibits *BRAF* mutations, and SW480 has *KRAS* mutations, representing common genetic alterations in colon cancer, which enhances the relevance of these models for disease research. Extracts from *Ferula* species, known for their high contents of biologically active compounds such as sesquiterpene coumarins, including conferone, have shown promising anticancer properties. Studies have highlighted the ability of conferone to inhibit P‐glycoprotein bioavailability and suppress cell proliferation through the generation of free radicals in colon cancer cells (HT‐29) [22]. These findings strongly suggest conferone as a potential candidate for developing anticancer drugs targeting CRC.

Conferone treatment caused significant inhibition of the expression and phosphorylation of FAK. Given the high expression of FAK in cancer cells, targeting FAK inhibitors represents a promising strategy for cancer therapy [23, 24]. Molecular docking and dynamics simulations indicated that conferone shares binding sites with known FAK inhibitors, suggesting a mechanism whereby conferone inhibits FAK activity. Inhibition of FAK phosphorylation prevents its binding with GRB2, thereby disrupting downstream signalling cascades, including phospho‐c‐Raf. This cascade inhibition effectively halts the activation of c‐Raf. Furthermore, FAK signalling influences the expression levels of c‐Myc, a multifunctional transcription factor pivotal in glutaminolysis. c‐Myc promotes GLS1 expression by inhibiting miR‐23a/b expression [25] and activates GLUL expression through GLUL promoter demethylation [26]. GLS1 catalyses glutamate synthesis from glutamine, while GLUL catalyses the reverse reaction. Conferone's inhibition of FAK not only reduced the c‐Myc levels and downregulated glutamine metabolism by suppressing GLS1 and GLUL expression but also reduced glutamate levels. This led to an increase in α‐KG production, potentially contributing to cellular oxidative stress and cell death [27]. Dual inhibition of FAK and glutamine metabolism by conferone makes the compound a promising anticancer agent.

Our study supports the hypothesis that conferone inhibits FAK expression, potentially reversing the EMT process [28] and disrupting the c‐Raf signalling pathway. This disruption suppresses c‐Myc activity, thereby inhibiting both glutamine anabolism and catabolism. Additionally, increased GLUD activity further boosts α‐KG production, potentially enhancing cellular reactive oxygen species generation and suppressing the glutaminolysis process (Figure 5). These multifaceted effects highlight the potential of conferone as a therapeutic agent against CRC. However, limitations exist. For instance, in vitro results may not translate perfectly to in vivo models. Further research is needed to explore its efficacy in a broader range and assess potential side effects. Additionally, while the mechanisms involving FAK and glutaminolysis are promising, their long‐term effects and potential for resistance require further investigation.

The present study includes limitations, as all the experiments were conducted in vitro using established CRC cell lines, and the anti‐cancer effects and efficacy of conferone have not yet been validated in vivo. Future studies employing animal tumour models will be essential in understanding and examining conferone's in vivo efficacy and pharmacokinetic properties. Additionally, we only focused on a subset of 37 compounds from our previously screened library and publications; therefore, a broader, unbiased screen may reveal additional candidates with anti‐CRC potential.

## 5 Conclusion

Our findings suggest that conferone, acting as an FAK inhibitor and reducing cancer proliferation, holds promise as an effective lead compound for future CRC treatment research. The targeted interaction model with FAK can also be utilised to screen and predict other natural compounds for potential anti‐cancer treatments. Among the 37 compounds tested, conferone demonstrated the strongest inhibitory effects against CRC cells; we plan to further optimise it to enhance its anti‐cancer activity. This study is expected to pave the way for the development of novel natural compounds useful for formulating CRC treatment strategies. To determine FAK as an anticancer approach in CRC therapy through glutamine metabolism reprogramming, further studies using a xenograft model will be carried out.

## Author Contributions

**Hien Thi My Ong:** methodology, data curation, investigation, formal analysis, writing – original draft. **Eda Ates:** investigation, software, formal analysis, visualization. **Min‐Jung Kang:** conceptualization, supervision, validation, funding acquisition, writing – review and editing. **Jaeyong Jung:** investigation, formal analysis, software. **Jae‐Chul Pyun:** validation, visualization, supervision. **Jeong Soo Sung:** investigation, software, formal analysis.

## Funding

This work was supported by the Ministry of Science and ICT, Republic of Korea (NRF‐2021R1A2C209370611), and Korea Institute of Science and Technology (2E33742).

## Disclosure

Submission declaration and verification: The manuscript has not been published previously.

## Conflicts of Interest

The authors declare no conflicts of interest.

## Supporting information


**Figure S1:** Effect of conferone treatment and PTK2 knockdown on the FAK–c‐Raf signalling axis.
**Table S1:** SiRNA sequences used for gene knockdown.

## Data Availability

The data that support the findings of this study are available from the corresponding author upon reasonable request.
