# Supplementary material for: Inhibitory Activity of Conferone on FAK Activity and Glutamine Metabolism in Human Colorectal Cancer
Source: J Cell Mol Med. 2026 Apr 29;30(9):e71165. doi: 10.1111/jcmm.71165 (PMC13128536; doi:10.1111/jcmm.71165)
Supplement: Supplementary file 1 — Figure S1: Effect of conferone treatment and PTK2 knockdown on the FAK–c‐Raf signalling axis. Table S1: SiRNA sequences used for gene knockdown. [file JCMM-30-e71165-s001.docx]

**Supplementary Materials of
Inhibitory activity of conferone on FAK activity and glutamine metabolism in human colorectal cancer**

Hien Thi My Ong^1,2^, Eda Ates^1,2^, Jaeyong Jung ^3^, Jeong Soo Sung ^3^, Jae-Chul Pyun^3^ and Min-Jung Kang^1,2^*

^1^Center for Advanced Biomolecular Recognition, Korea Institute of Science and Technology, 5, Hwarang-ro 14-gil, Seongbuk-gu, Seoul 02792, Republic of Korea

^2^Division of Bio-Medical Science & Technology, KIST School, University of Science and Technology, 5, Hwarang-ro 14-gil, Seongbuk-gu, Seoul 02792, Republic of Korea

^3^Department of Materials Science and Engineering, Yonsei University, 50 Yonsei-ro, Seodaemun-gu, Seoul 03722, Republic of Korea

*** Corresponding author**

Dr. Min-Jung Kang

Center for Advanced Biomolecular Recognition

Korea Institute of Science and Technology

Seoul 02792, Republic of Korea

Tel: +82-2-958-5088

E-mail address: [mjkang1@kist.re.kr](mailto:mjkang1@kist.re.kr)

***Supplementary Figure S1. Effect of conferone treatment and PTK2 knockdown on the FAK–c-Raf signaling axis***

A) Representative immunoblot analyses showing protein levels of FAK, phosphorylated FAK (p-FAK), c-Raf, and phosphorylated c-Raf (p-c-Raf) in cells subjected to PTK2 siRNA knockdown and/or conferone treatment.
(B) Densitometric quantification of the immunoblot signals from independent experiments. Band intensities were normalized to the loading control and expressed relative to growth control (GC).

GC, growth control; TC, transfection control; DMSO, vehicle control; Conf, conferone; PTK2 siRNA, PTK2-targeting small interfering RNA; Cont, siRNA knockdown continued; Disc, siRNA knockdown discontinued


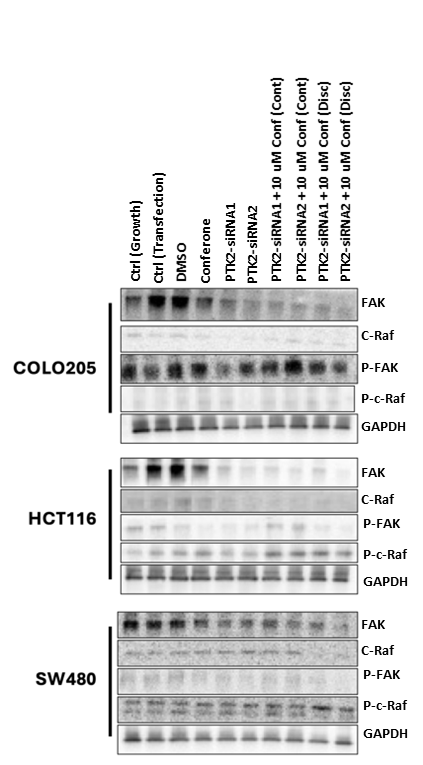


**B**

**A**

***Supplementary Table S1. SiRNA sequences used for gene knockdown***

|  | | |
| --- | --- | --- |
| **Name** | **Forward** | **Reverse** |
| **PTK2-1** | 5′-GUCUGUCUAGAAUCUCUGUtt-3′ | 5′-ACAGAGAUUCUAGACAGACtt-3′ |
| **PTK2-2** | 5′-CUGCAAUCCCACACACUCUtt-3′ | 5′-AAGAGUGUGGGGAUUGCAGtt-3′ |
|  |  |  |
| As control, AccuTarget™ Negative Control siRNA (Bioneer) was employed. | | |
